# Experimental testing of reciprocal effects of nutrition and parasitism in wild black capuchin monkeys

**DOI:** 10.1038/s41598-017-12803-8

**Published:** 2017-10-06

**Authors:** Ilaria Agostini, Ezequiel Vanderhoeven, Mario S. Di Bitetti, Pablo M. Beldomenico

**Affiliations:** 1 Instituto de Biología Subtropical (IBS), Universidad Nacional de Misiones (UNaM), Consejo Nacional de Investigaciones Científicas y Técnicas (CONICET), Puerto Iguazú (Misiones), Argentina; 2 Asociación Civil Centro de Investigaciones del Bosque Atlántico (CeIBA), Puerto Iguazú, Argentina; 3 Instituto Nacional de Medicina Tropical (INMeT), Puerto Iguazú, Argentina; 4 Facultad de Ciencias Forestales, UNaM, Argentina; 5 Laboratorio de Ecología de Enfermedades, Instituto de Ciencias Veterinarias del Litoral (ICiVet-Litoral), Universidad Nacional del Litoral (UNL)/Consejo Nacional de Investigaciones Científicas y Técnicas (CONICET), Esperanza, Santa Fe, Argentina

## Abstract

Nutritional stress may predispose individuals to infection, which in turn can have further detrimental effects on physical condition, thus creating an opportunity for reciprocal effects between nutrition and parasitism. Little experimental investigation has been conducted on this “vicious circle” hypothesis in wild animals, especially under natural conditions. We evaluated the reciprocal effects of nutritional status and parasitism using an experimental approach in two groups of wild black capuchin monkeys (*Sapajus nigritus*). Across two consecutive winters, we collected faecal samples from identified capuchins to determine presence and load of gastrointestinal helminthes, and measured individual body mass as a proxy of physical condition. Food availability was manipulated by provisioning monkeys with bananas, and parasite burdens by applying antiparasitic drugs to selected individuals. We found no effect of antiparasitic drugs on physical condition, but parasite loads decreased in response to high levels of food availability. Our results represent the first experimental evidence that the nutritional status may drive parasite dynamics in a primate.

## Introduction

Understanding the interacting dynamics of nutrition, parasitism and host health status is crucial for predicting changes in disease patterns and developing conservation plans for endangered species^1,2^. One of the variables that may affect these dynamics is food availability. When food supply is low animals may be more prone to infections^1^. This association is likely due to trade-offs between costly immune defences and other competing energy-demanding processes, such as homeostasis, growth and reproduction^3,4^. An animal facing a reduction of nutrient availability may be forced to modify its physiological economy, which may affect investment in immune defences^5^, and consequently its susceptibility to diseases^6–8^.

Parasites can be detrimental to host’s health. By extracting the host’s resources and/or inducing a nutritionally demanding immune response, even relatively benign parasites seem likely to exert a negative effect on the fitness of hosts (e.g. cowpox virus in *Microtus agrestis* L.^9^). Quantitative disease studies have proved to be challenging, mainly due to the difficulty in obtaining appropriate samples^2^. However, gastrointestinal parasites represent an exception, since it is possible to diagnose them through non-invasive analysis of faecal samples^10^. These parasites can affect host fitness either directly through their pathological effects, or indirectly by deteriorating physical condition^11,12^. Nevertheless, if the host is in good nutritional condition, it might be able to allocate more resources to counter and limit parasitic infections and/or their damage, as a result of proficient defences. On the other hand, a host in poor nutritional condition can show a weaker immune response against infections, which in turn become more likely and of higher intensity^1,4,13,14^. These reciprocal effects between condition and infection could trigger a “vicious circle”, where host health becomes increasingly deteriorated and severe infection more likely, and so on^1,6,15,16^. A few field experimental studies manipulating both parasite infections and food availability have tested this hypothesis. For example, contribution of parasites to periodic population crashes has been demonstrated in the multiannual cycling Soay sheep (*Ovis aries*) in the St. Kilda archipelago, Scotland. Experiments involving helminth removal as well as artificial infection provided evidence that parasitism contributes to mortality in malnourished sheep, exacerbating the effects of food shortage^17^. Similarly, an experiment where replicated populations of white-footed mice (*Peromyscus leucopus*) and deer mice (*Peromyscus maniculatus*) were treated with antihelminthic drugs and/or food supplementation, showed that each treatment had a significant effect on mice survival when administered alone, but both combined had an impact much greater than the sum of each individual effect^18,19^. A more recent experiment in voles showed that populations with food supplementation and not exposed to *Bordetella bronchiseptica* infections exhibited consistent positive population growth in late winter. In contrast, populations experimentally infected with *B. bronchiseptica* and/or without food supplementation declined in size^20^. These consistent results strongly suggest a synergy between infection and nutritional condition leading to the operation of a vicious circle.

Data from observational epidemiological studies in humans also support this notion^21,22^, but so far, there is no experimental evidence of these dynamics in primates. Given their phylogenetic relatedness, wild populations of non-human primates provide an ideal model for testing hypotheses on infectious disease-nutrition dynamics in nature, allowing inferences on the impact of pathogens in human populations^2^. A few studies have tackled this issue in non-human primates using an observational or quasi-experimental approach. Evidence of effects of food availability and parasite infection on population abundance have been found for red colobus monkeys (*Piliocolobus tephrosceles*) living in forest fragments. A decline in food availability was linked to effects that indicated a direct negative impact on colobus population abundance, and an indirect effect via parasite infections^23^. Another study on black howler monkeys (*Alouatta pigra*) in a hurricane damaged forest fragment in Belize found similar results: both fruit consumption and multispecies parasite infections were associated with howler population abundance^24^. Finally, in an effort to explain the broad mortalities of mantled howler monkeys (*Alouatta palliata*) due to botfly infections in Panama, researchers inferred that mortality peaks were due to a combination of factors including both the physical condition of monkeys and larval burdens of parasitised individuals, which were especially critical during periods in which the population was facing nutritional stress^25^.

Black capuchin monkeys (*Sapajus nigritus*) are medium sized (2.5–3.6 kg^26^) diurnal Neotropical primates. Their omnivorous diet, based on fruits and arthropods^27^, potentially exposes them to a greater variety of parasites compared to other primates, for example those that feed predominately on leaves^28^. In addition, *Sapajus nigritus*, which is endemic to the Atlantic Forest, approaches its southern geographic boundary in the province of Misiones, Argentina, where it has to endure a marked seasonality in food availability^29^. These characteristics make this southern capuchin population an excellent model to test the “vicious circle” hypothesis. Besides, in Iguazú National Park, the possibility of simultaneous manipulation of food resources and parasitic infection enables dissecting the role of each of these two variables in determining the physical condition and parasitism dynamics in wild capuchins.

In this study, we manipulated food availability at the group level (high vs. low provisioning regime), and parasite burden at the individual level (antiparasitic drugs administration to selected subjects within groups), switching treatment between groups and among individuals across two consecutive winters. We used measures of parasite output in faeces as proxies of parasitism, as well as repeated measures of body mass as an index of individual physical condition^30^. According to the “vicious circle” hypothesis, by jeopardising host defences, nutritional stress makes individuals more prone to parasite infection^31,32^, which in turn can have further detrimental effects on physical condition^1,18^. Based on this hypothesis, we expect nutrition and parasitism to show reciprocal effects. In particular we predict that (1) capuchins will show lower parasitism when subjected to high food provisioning compared to low food provisioning, and that (2) individuals will increase their body mass when they are treated with antiparasitic drugs than when they are not treated.

## Results

We identified eight parasite species from the 687 faecal samples collected from 30 capuchins belonging to two study groups (Macuco and Spot) during two consecutive winters (Supplementary Table S1). We recovered parasites from 21 of 24 adult/subadult individuals (87.5%) in the winter of 2013, and 23 of 25 individuals (92.0%) in the winter of 2014. These included six types of nematodes [*Strongyloides* sp. (Family Strongyloididae), *Trichuris* sp. (Family Trichuridae), *Ascaris* sp. (Family Ascarididae), an undetermined Subuluridae, an undetermined Spiruridae, and *Filariopsis* sp. (Family Filaroididae)], a cestode belonging to the family Hymenolepididae, and an undetermined trematode. The most prevalent parasites among adults and subadults in both capuchin groups were *Filariopsis* sp. (prevalences of 54 and 72% in 2013 and 2014, respectively) and Hymenolepididae (58–64%), followed by *Strongyloides* sp. (20–48%). The other parasite species had consistently lower prevalences across the two winters (0–12%) (Supplementary Table S2). In addition, we found oocysts of undetermined protozoan species (probably an Apicomplexan) which accounted for an overall prevalence of 24.0–41.7%.

We obtained 46 repeated measures of body mass from 11 adult individuals (7 from Macuco group and 4 from Spot) in winter 2013, and 45 measures from 13 adults (8 from Macuco group and 5 from Spot) in winter 2014 (Supplementary Tables S1–S3). Due to changes in group composition across the two winters (Supplementary Table S4), as well as different proneness to approach the scale among individuals, we obtained body mass measures for both winters for six females and three males.

**Figure 1:**
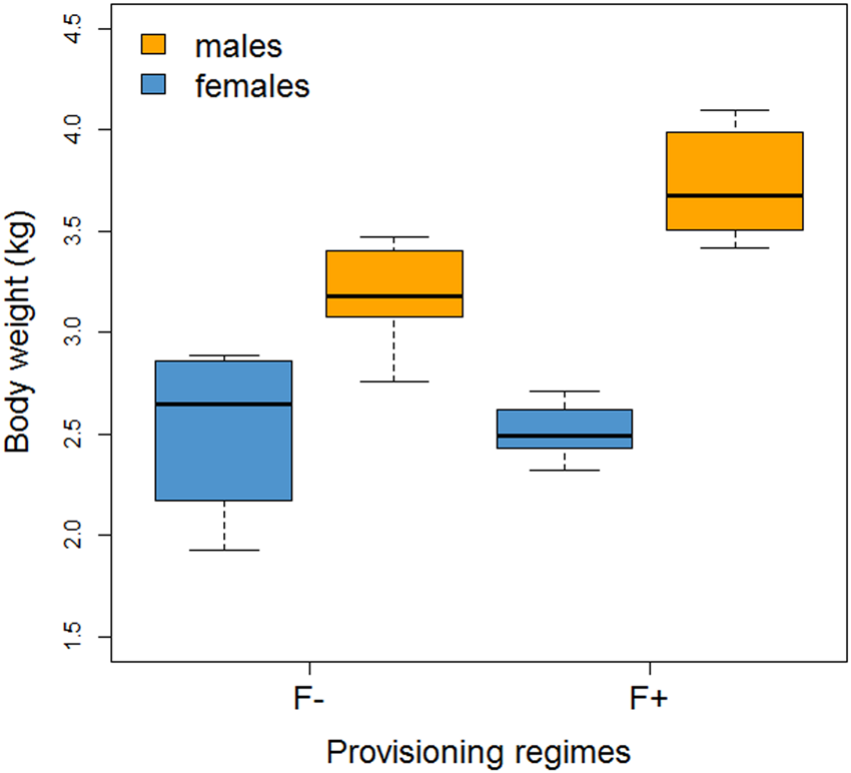
Individual body mass between different provisioning regimes, differentiated according to sex. The two food provisioning levels were: high provisioning (F+) and low provisioning (F−). Blue boxes denote adult/subadult females, while orange boxes represent adult/subadult males. Horizontal lines show the medians. Bottom and top of the box show the 25^th^ and 75^th^ percentiles and vertical dashed lines (whiskers) the ranges.

As an evidence for the effectiveness of our experimental manipulations, we found that the antiparasitic treatment reduced infection. The treatment reduced load of *Filariopsis* sp. larvae (first-ranked univariate model), and contributed to reduce egg load of the Hymenolepididae (second-ranked univariate model) (Supplementary Table S5). When individuals were treated they had approximately half the odds (odds ratio = 0.55) of being infected by *Filariopsis* sp. than when not treated. The importance of antiparasitic treatment effect on this parasite is also evident from model selection according to Akaike weight (Table 1), and its relative importance value (i.e. the sum of the Akaike weights for a predictor over all models in which it occurs, 0.60) (Table 2). The antiparasitic drug’s effect on the probability of being infected by the Hymenolepididae was much lower as evidenced by the relative importance value (0.23) (Tables 1 and 2). However, importantly, the treatment had a strong effect reducing parasite richness by 94% (relative importance = 1.00; all models fitted controlling for faecal sample’s weight; Tables 1 and 2). In addition, we found that provisioning affected individual body mass: higher provisioning with bananas resulted in an 8% increase in body mass (provisioning appeared in the two top-ranked models, with an importance value of 0.98, all models fitted controlling for individual sex; Tables 3 and 4). However, this effect was more evident for males than for females (Fig. 1), probably because of males’ greater capacity to monopolise concentrated food resources such as provisioning platforms.

**Table 1:** Model selection on the basis of second-order Akaike Information Criterion (AICc), explaining variation in (a) *Filariopsis* sp. infection, (b) Hymenolepididae infection, (c) parasite richness, and (d) probability of multiple infections.

Response variable	Model	K	ΔAICc	AICc weight
**(a)** ***Filariopsis*** **infection**	Provisioning + Antiparasitic + Faecal sample weight^†^	6	0.00	0.39
	Provisioning + Faecal sample weight	5	1.11	0.22
	Antiparasitic + Faecal sample weight	5	1.85	0.16
	Faecal sample weight	4	1.98	0.15
	Null model	3	6.07	0.02
**(b) Hymenol. infection**	Provisioning	4	0.00	0.32
	Null model	3	1.03	0.19
	Provisioning + Antiparasitic	5	1.65	0.14
	Provisioning + Faecal sample weight	5	2.02	0.12
	Antiparasitic	4	2.93	0.07
	Fecal sample weight	4	3.01	0.07
	Provisioning + Antiparasitic + Faecal sample weight^†^	6	3.66	0.05
**(c) Parasite richness**	Provisioning + Antiparasitic + Faecal sample weight^†^	6	0.00	0.60
	Provisioning + Antiparasitic	5	0.79	0.40
	Null model	3	7.31	0.01
**(d) Multiple infections**	Provisioning	4	0.00	0.39
	Provisioning + Antiparasitic	5	0.94	0.24
	Provisioning + Faecal sample weight	5	1.81	0.16
	Provisioning + Antiparasitic + Faecal sample weight^†^	6	2.80	0.10
	Null model	3	3.78	0.06

^†^Global model. The null model, the global model and selected models with strong support (95% cumulative weight criteria) are provided. Models are listed in decreasing order of importance. For all models random effects were Individual identity nested in Group (See model outputs in Supplementary Material).

**Table 2:** Results of full Generalised Linear Mixed Models (GLMMs) including all fixed factors of interest for each response variable.

Response variable	Explanatory variable	Parameter likelihood	Parameter estimate ± SE	Confidence interval	
				Lower	Upper
**(a)** ***Filariopsis*** **infection**	Intercept	—	−5.894 ± 1.317	−8.874	−3.602
	Faecal sample weight	1.00	0.890 ± 0.414	0.178	1.840
	Provisioning (F−)	0.67	0.597 ± 0.306	0.003	1.198
	Antiparasitic (A−)	0.60	0.595 ± 0.348	−0.060	1.312
**(b) Hymenol. infection**	Intercept	—	−3.017 ± 0.916	−5.030	−1.266
	Provisioning (F−)	0.63	0.572 ± 0.308	−0.046	1.204
	Antiparasitic (A−)	0.23	0.207 ± 0.324	−0.425	0.888
	Faecal sample weight	0.21	0.046 ± 0.282	−0.484	0.672
**(c) Parasite richness**	Intercept	—	−2.562 ± 0.539	−3.701	−1.576
	Provisioning (F−)	1.00	0.445 ± 0.153	0.148	0.748
	Antiparasitic (A−)	1.00	0.290 ± 0.180	−0.052	0.654
	Faecal sample weight	0.60	0.278 ± 0.176	−0.043	0.650
**(d) Multiple infections**	Intercept	-	−4.070 ± 1.292	−7.048	−1.840
	Provisioning (F−)	0.94	1.166 ± 0.491	0.249	2.211
	Antiparasitic (A−)	0.36	0.552 ± 0.566	−0.466	1.813
	Faecal sample weight	0.27	−0.179 ± 0.408	−0.888	0.762

Levels of categorical predictors not included in the intercept are the following: Low Provisioning (F−), and Antiparasitic Not Treated (A−). The reference levels included in the intercept are High Provisioning (F+), and Antiparasitic Treated (A+).

**Table 3:** Model selection on the basis AICc explaining variation in individual body weight.

Response variable	Model	K	ΔAICc	AICc weight
**Body weight**	Provisioning + Sex	6	0.00	0.73
	Provisioning + Antiparasitic + Sex^†^	7	2.27	0.23
	Null	4	21.23	0.00

^†^Global model. The null model, the global model and selected models with strong support (95% cumulative weight criteria) are provided. Models are listed in decreasing order of importance. For all models random effects were Individual identity nested within Group (See model outputs in Supplementary Material).

**Table 4:** Results of full GLMM including all fixed factors of interest for individual body weight variation.

Response variable	Predictor and control factors	Parameter likelihood	Parameter estimate ± SE	Confidence interval	
				Lower	Upper
**Body weight**	Intercept	—	2.585 ± 0.151	2.290	2.880
	Sex (m)	1.00	1.111 ± 0.133	0.850	1.371
	Provisioning (F−)	0.96	−0.209 ± 0.068	−0.341	−0.076
	Antiparasitic (A−)	0.24	−0.040 ± 0.068	−0.174	0.093

Levels of categorical predictors not included in the intercept are the following: Low Provisioning (F−), and Antiparasitic Not Treated (A−), and males (m) for Sex. The reference levels included in the intercept are High Provisioning (F+), Antiparasitic Treated (A+), and females (f) for Sex. Parameter likelihoods (i.e. relative importance of explanatory variables), estimates (±SE) and 95% confidence interval for the parameters of explanatory variables describing variation in individual body weight. See methods for details. Explanatory variables are listed in decreasing order of importance (See model outputs in Supplementary Material).

**Figure 2:**
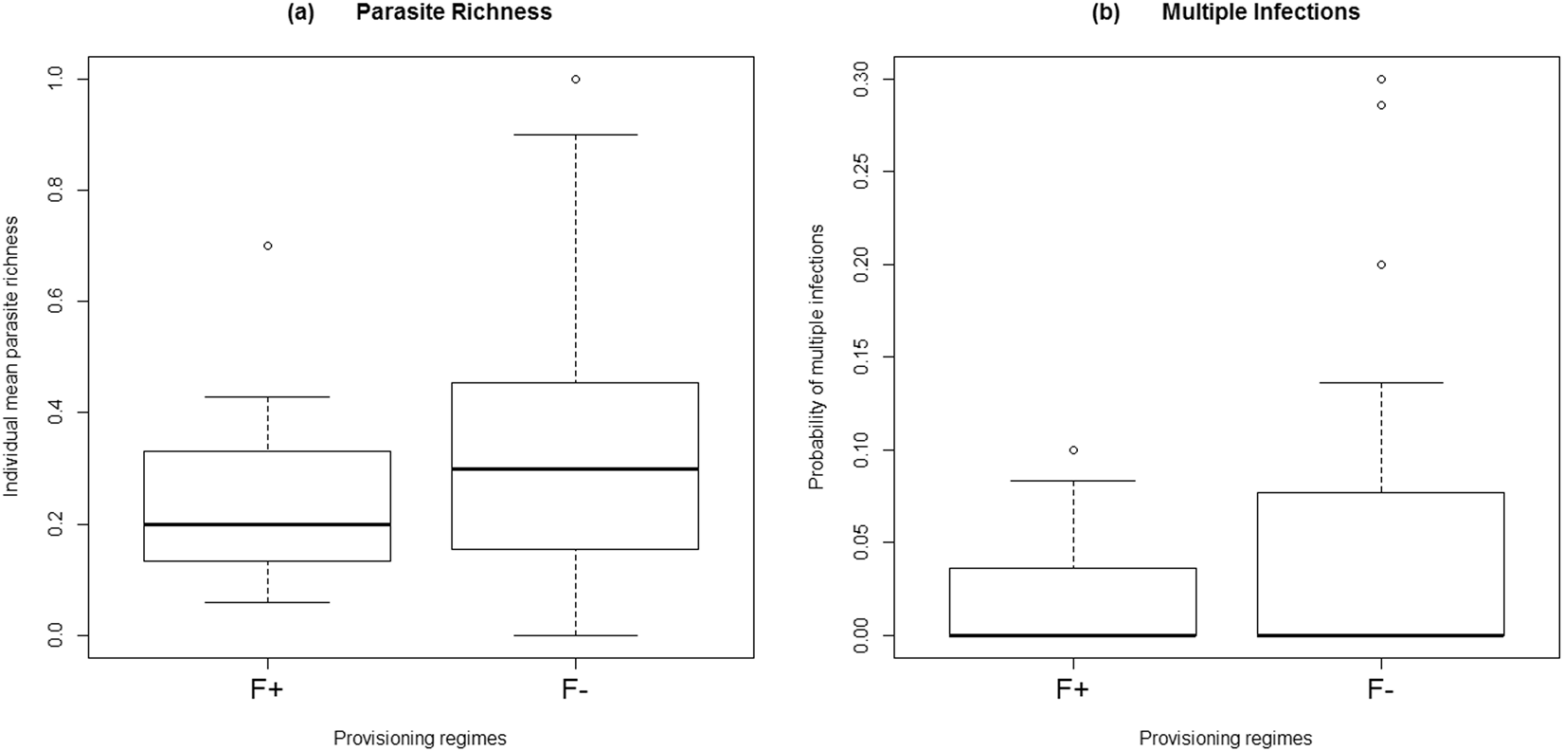
(**a**) Individual mean parasite richness and (**b**) probability of multiple infections between different regimes of provisioning. The two food provisioning levels were: high provisioning (F+) and low provisioning (F−). Horizontal lines show the medians. Bottom and top of the box show the 25^th^ and 75^th^ percentiles and whiskers show the largest data point that is less than 1.5 times the interquartile range above the 75^th^ percentile. Circles indicate outliers.

Model selection on the basis of second-order Akaike’s Information Criterion (AICc)’s weights revealed a high importance of provisioning for parasitism. The risk of *Filariopsis* and Hymenolepididae infection in individuals in high provisioning regime was halved (odds ratios = 0.55 and 0.56, respectively) when compared to capuchins receiving low provisioning. The importance of provisioning effect on these parasitic infections is also evident from model selection according to Akaike weight (Table 1), and relative importance values (0.67 and 0.63, respectively) (Tables 1 and 2). In addition, higher provisioning had a strong and significant effect in decreasing parasite species richness and the probability of suffering multiple infections compared to lower provisioning (effect sizes = 95% and 99%, respectively; provisioning in the first top-ranked models with an importance of 1.00 and 0.94, respectively; Fig. 2a,b; Tables 1 and 2). On the other hand, the antiparasitic treatment had a negligible effect on body mass (an increase of just 1.5%; importance value = 0.24, Tables 3 and 4). Even when considering those groups that were under a low provisioning regime, the best model explaining individual body mass included only sex; the antiparasitic treatment did not improve the explanatory power (weight ~ sex; d.f. = 5, AICc weight = 0.81; weight ~ sex + antiparasitic treatment; d.f. = 6, AICc weight = 0.19).

## Discussion

To the best of our knowledge, this is the first study to experimentally evaluate the reciprocal effects of nutrition and parasitism in a primate species. Our results matched one of the predictions of the “vicious circle” hypothesis: higher levels of food provisioning contributed to decrease the probability of infection with the two most common parasites, a nematode (*Filariopsis* sp.) and a cestode (Hymenolepididae), as well as the probability of presenting multiple infections and overall parasite richness. However, although the antiparasitic treatment effectively reduced parasite loads, we could not demonstrate that parasitism affected the physical conditions of the capuchins in our study.

Although infections with parasites have been frequently studied as a dichotomous variable, when a host is exposed to a pathogen there is a continuum of possible outcomes. The specific outcome depends on parasite’s pathogenicity and infective dose, but also on the integrity and strength of host’s defences against the pathogen^1^. These defences include two components: resistance (e.g. antigen-specific immune response), which enables host to limit parasite burden, and tolerance, which is the ability to minimize the damage caused by a given parasite burden^4,33^. The relative extent of resources allocated to defences would depend on nutrient availability and the trade-offs between defence efforts and other nutrient-demanding processes such as growth, reproduction, and thermoregulation^3–5^, and here we offer evidence of that in wild monkeys. When a capuchin is well nourished, its immune defences are strong and functional, and consequently it will be more resistant to parasitic infections, compared with an individual that is poorly nourished (a condition which is common during winter at our study site^34^).

Of the two parasites most frequently found in capuchins, the Hymenolepididae has probably an indirect life cycle (although one species, *Hymenolepis nana*, has a direct cycle involving no intermediate host)^28^. Taking into account our current knowledge on the Hymenolepididae of Cebidae, the intermediate host most likely is a beetle^35^. Therefore, this parasite is probably acquired by monkeys during foraging, as invertebrates are among the most important food items in a capuchin’s diet^36^. If eating more bananas would imply a decreasing consumption rate of invertebrates (including the infected beetles), a plausible alternative explanation could be that provisioning caused a change in the feeding behaviour and a consequently resulted in a decreased exposure to the intermediary host of this parasite, and not necessarily is the result of an improvement in capuchins’ defences^37^. However, a study examining how crop raiding and food provisioning affect diet and foraging activities in a related capuchin monkey species (*Cebus capucinus*) found that, although anthropogenic fruits increased the overall proportion of fruits in human-commensal capuchins’ diet, the rate of insect consumption was similar to that of wild-foraging groups^38^. In addition, at our study site we observed that capuchins do not cease to forage on invertebrates despite their banana supplement. In the case of *Filariopsis* sp., even though its life cycle is yet to be described, there is evidence strongly suggesting that this nematode has a direct cycle, not including any intermediate hosts. In fact, given the relative high prevalence of this parasite in capuchins, and the phylogenetic relatedness with *Filaroides* spp., which have a direct life cycle and immediately infective larvae passed in host’s faeces, it is likely that *Filariopsis* can also be directly transmitted^39,40^. Thus, in this case, the alternative explanation is even less plausible.

Turning to tolerance, the lack of an effect of the experimental reduction of parasites on capuchins’ physical condition suggests that these monkeys are tolerant to the existing parasite burdens^33^. Nonetheless, an effect of parasites on the condition of monkeys should not be ruled out, considering (1) the incomplete parasite removal achieved; (2) the moderate to low burdens that prevailed during the experiment; and (3) the relatively low statistical power. In particular, we acknowledge the low statistical power as the main limitation of our study. In a natural context, it is extremely difficult to obtain large numbers of individual body mass repeated measures during two consecutive time periods. However, we were interested in effects of high biological magnitude that could be detectable with limited statistical power. Our failure to find effects (i.e. antihelminthic treatment not enhancing body weight) might be due to lack of statistical power, but probably it is reflecting the low burdens of parasites this primate population had during the study period. Although we failed at finding it in our study subjects, the effect of parasites on physical condition has been extensively documented in animals^32,41–43^, and may even affect other aspects of host fitness. For example, experimental deworming of wild vervet monkeys (*Chlorocebus aethiops*) has shown that a decrease in parasite burdens could affect an individual’s condition not only by directly increasing its body mass, but also indirectly, through a change in host’s activity patterns (i.e. decreasing resting and increasing traveling time), and social relationships (i.e. increasing the number of nearest neighbours individuals and the frequency of social interactions), that could in turn affect reproduction and fitness^44^. Besides all of the reasons mentioned, the lack of effect of the parasite-reduction treatment on individual body mass even in the more food-limited group could indicate that the impact of the parasite burdens affecting our capuchins is negligible, at least under the relatively benign environmental conditions that prevailed during the study period.

Among studies examining the potential interaction between food availability and parasitism, evidence in support of reciprocal effects were obtained for species with multiannual population density cycles that periodically suffer a population decline and thus may face food shortage^18^, as well as in non-cycling species^15,17,23,45^. Although the interactive effects of food stress and parasitism were potentially responsible for the decline of the red colobus monkeys populations in African rain forest fragments^23^, these effects were not observed in continuous forest, where food resources were abundant for the same species^46^. Therefore, different conditions (e.g. levels of food stress) may or may not promote an interaction between nutritional status and parasitic infections in an animal population. It has been argued that the interaction will only occur under extreme conditions, i.e. when individuals suffer from high levels of nutritional stress^6,46^. Although capuchins at Iguazú generally face a period of food shortage during winter (June – August), due to seasonal decrease in fruit and arthropod availability^29^, resource supply may not have reached the critical low threshold required to trigger “vicious circle” dynamics. Likely, both food availability and exposure to parasites vary from year to year, thus capuchins may occasionally face more severe winter conditions that could promote a synergistic interaction between these two factors.

Alternatively, or in parallel, the lack of complete evidence for reciprocal effects leading to “vicious circle” dynamics could be due to the relatively low parasite loads of capuchin monkeys at our study site. In fact, if individuals were infected with low burdens of few parasites with relatively low pathogenicity, parasitism would not be expected to be important enough to trigger a significant change in physical conditions. The predominant parasites found in our study are known to have low pathogenic effects at low to moderate burdens^40,47,48^. Thus, it is likely that the reduction of the existing parasite burdens could not translate into a significant improvement in individual physical conditions, at least not large enough to be detected as body mass changes.

In conclusion, we succeeded in demonstrating the boldest prediction of the “vicious circle” hypothesis: the nutritional status of capuchins drives the dynamics of some of their parasites. On the other hand, at the levels of parasitism recorded, capuchins appear to tolerate these parasites. Future research should be aimed at evaluating these processes under circumstances of more severe food limitation and greater parasite exposure. In addition, future experimental studies with wild primates should put effort in obtaining greater sample sizes to increase statistical power and be able to test for synergistic effects of food availability and parasitism.

## Methods

### Study site and subjects

The field work was carried out in Iguazú National Park, Argentina (25°40′S; 54° 30′W), in the south-western border of the Upper Paraná Atlantic Forest. This semi-deciduous forest is characterised by a humid subtropical climate with a marked seasonality in day length and temperature^49^. Mean annual rainfall in the area is 2000 mm and precipitation is evenly distributed throughout the year^50^. There is a seasonal variation in the production of fleshy fruits and arthropods, which is lower during winter months (June-August) and reaches its maximum between October and January^51^. Due to this seasonal pattern, winter is a critical time during which this black capuchin population depends mostly on bamboo shoots (*Chusquea ramosissima*) and meristems and leaf bases of epiphytes (orchids, bromeliads and *Philodendron*)^34^. Black capuchins live in polygamous multi-male multi-female groups of 7–30 individuals, in which the alpha-male has a central role and females, which are philopatric, establish a linear dominance hierarchy^34^. Individuals can be identified on the basis of physical characteristics, such as facial colour patterns, body size and shape of tufts^29^.

Iguazú hosts a stable population of capuchins in a continuous and well preserved forest. Within the park, researchers have identified and habituated individuals of five groups. These individuals have been monitored continuously since 1991^52^. Every winter (June to August), the season of food scarcity, one or two of the groups that have been studied for over 25 years in Iguazú National Park, are provisioned by researchers with bananas on feeding platforms^52^. The banana is a nutritious and easily digestible fruit, which has become a staple food and main energy source for many human populations^53^. It is a highly preferred food by capuchins at our study site, as evidenced, among other behaviours, by the high rate of food-associated calls produced when feeding on them^54^. The opportunity of provisioning monkeys with bananas during winter allows the experimental manipulation of food availability in the most critical time of the year, when fruits and arthropods are scarce^51^ and thus, when animals that do not receive an extra food provision would likely suffer a nutritional stress. In addition, the high degree of habituation of capuchins at the site enables manipulating the level of parasitism by supplying antiparasitic drugs to selected individuals.

### Behavioural data and faecal samples collection

During four months (May-August) in winter 2013 and four months in winter 2014, we collected data on the behaviour of adult/subadult individuals belonging to two groups, Macuco (10–19 adult/subadults out of 23–27 individuals) and Spot (10–12 adult/subadults out of 17–21 individuals). We classified females as adults if they were older than five years, and subadults if they were four to five years old (Supplementary Table S4). This age in capuchin females generally corresponds to their first estrous with conception^52^. However, we had two females who conceived earlier, at five years of age, and we classified them as adults. We classified males as adults when older than six years, which commonly coincides with their emigration from the natal group^52^; and subadult when five to six years old. In most cases, we knew the age of each individual with a precision of one to 30 days. In fact, researchers working on capuchins at Iguazú keep long-term records of all births, migrations and deaths in the study groups. Group composition slightly changed across the two years (Supplementary Table S4). The team followed them during 108 days during 2013 and 107 days during 2014, from dawn to dusk (approx. 12 hs/day). In a parallel study, Barbara Tiddi and Brandon Wheeler collected and analysed data on all occurrences of dyadic agonistic interactions (aggression, submission and spatial displacement) that involve adult individuals in each group in the context of a provisioning experiment with the objective of establishing the dominance hierarchy (Tiddi and Wheeler, unpublished data). In order to determine the reproductive status of adult females, we distinguished lactating females with dependent infants (<1 year old) from non-lactating ones.

To obtain data on parasitism, we collected faecal samples opportunistically from identified adult and subadult individuals of both groups obtaining a total of 231 faecal samples from 24 individuals during winter 2013 (mean ± SD samples per individual: 9.62 ± 3.82), and 456 faecal samples from 25 individuals during winter 2014 (18.24 ± 10.12) (Supplementary Table S1). For the analysis of gastrointestinal parasites, a portion of approximately 5 g of each faecal sample was stored in a solution of 10% formalin. To take into account the potential variability in parasite shedding within winter months, we put a lot of effort in collecting multiple monthly faecal samples from each individual.

### Parasitological technique

Faecal samples were analysed in the Laboratory of Parasitology of the National Institute of Tropical Medicine (INMeT) in Puerto Iguazú, Misiones, Argentina. We processed samples using a semi-quantitative flotation method with a saturated sugar solution^55^. We weighted 3 g of faecal sample, then homogenised it, and after centrifugation we mounted it on a slide. We used a Carl Zeiss Primo Star Microscope to identify parasite structures and took pictures with Carl Zeiss AxioCam Cc1 using the 40x magnifier. Eggs and larvae were counted and identified on the basis of colour, shape, content and size. Although most samples reached 3 g after being processed, some samples did not reach this threshold. However, since some parasites were detectable even in < 3 g samples, instead of discarding them, we decided to consider faecal sample’s weight as a control factor in our analyses.

### Capuchins’ physical condition

We used capuchin individuals’ body mass as a proxy for physical condition. To measure the body mass of each adult and subadult individual we mounted an electronic platform scale on a tree trunk using a metal and wooden stand (a similar technique has been already used in wild capuchins^56^). The stand was composed of a wooden platform (40 × 45 cm) attached to a vertical support (80 cm) that was tight to the trunk by ropes at 1.2–1.3 m above the ground. The electronic scale (EOB15K5 – Kern, 15 kg model, sensitivity to 5 g; weighing plate’s dimensions: 31.5 × 30.5 × 0.5 cm) was laid on the metal support. A metal bowl was inserted into a metal collar fixed to the distal portion of the platform’s support, at a distance of 5 cm from the scale. We provisioned the bowl with 1–2 smashed banana pieces. The weight was displayed on a digital screen attached to the scale via a 200 cm long cable. The observer stood about 2 meters from the scale holding the digital display and recording the weight when the subject was stationary and fully supported by the scale as the capuchins visited the provisioned bowl (Supplementary Fig. S1a). The scale was frequently set in proximity of a provisioning platform site (see below) to increase the chance of being detected by monkeys. We put an effort in standardising our weighing procedures across different individuals: all subjects approaching the scale ingested the piece of banana used as bait (i.e. we minimised the potential bias associated to differential food ingestion among individuals before being weighed). Besides, capuchins show a relatively fast rate of passage of nutrients and waste products through their digestive tract compared, for example, with more folivorous, bulk feeding primates such as howler monkeys^57^. This results in a less variable and relatively smaller volume of food contained in the digestive tract of a wild capuchin at any moment when compared to a folivorous monkey species. Therefore, although capuchin monkeys may increase temporarily their body weight immediately after food consumption, the relatively small gut content, fast rate of food ingestion and passage through the digestive tract, and thus the short retention time, makes weight fluctuations likely to be negligible. Most individuals were weighed repeated times during each field season in order to obtain reliable average body mass values.

### Experimental protocol

We manipulated food availability by provisioning capuchins with bananas and gastrointestinal parasite load by supplying them with antiparasitic drugs. Both groups were provisioned during winter, but with different regimes. While one group was supplemented with high provisioning (3 bananas/platform × 3 platforms/site × 3 sites = 27 bananas), the other group received low provisioning (2–3 bananas/platform × 1 platform/site × 1–3 sites = 2–9 bananas). The low provisioning regime was established because it was part of a parallel experiment conducted by B. Wheeler and B. Tiddy on the same study groups. Although ideally we would have preferred to provide no food at all, a slight provisioning functioned as a control, reducing the chances that potential differences in parasites found in individuals from the two study groups might have been attributable to consumption of a food item (i. e. bananas) by one group, but not the other. Provisioning followed a protocol which has been used with capuchin groups in Iguazú repeatedly for over 20 years^58,59^. We provided bananas distributed across one to three sites (according to the regime), approximately 250 m apart one from another. Within each site, we presented bananas on one or three (according to the regime) 1 × 1 m wooden platforms hanging from tree branches (Supplementary Fig. S1b). When three platforms were present at a site, they were set 15 m apart in order to ensure that no individual could monopolise more than one platform at a time^51^. Before raising the platforms up on the tree, we cut each banana into pieces of approximately 2.5 cm and spread them evenly on the platform’s ground.

To reduce gastrointestinal parasite load of some selected individuals (approximately a half of adults/subadults each year) within each group, we supplied them with antiparasitic drugs. We used a cocktail of ivermectin and praziquantel. The first drug reduces infections by nematodes and ectoparasites, while the second removes cestodes. The two are considered non-toxic and well-tolerated drugs which are frequently used with monkeys in captive conditions^60–62^, and have been tested also in wild primates^63^ and small mammals^18^. We prepared the antiparasitic cocktail by mixing 0.6–0.8 mg of ivermectin (Sistema ENDECTOCIDA^®^ 5 mg – Holliday Scott Laboratory; recommended dose: 0.2 mg/kg body weight^64^) with 15–20 g of praziquantel (CESTODAN^®^ 25 mg – König Laboratory; recommended dose: 5 g/kg body weight^64^), depending whether we were supplying a female (lower dose) or a male (higher dose). We stuffed the powder mix into a 5 cm-piece of banana by previously digging a little hole on one extremity, filling it with the powder and covering it by spreading the extremity of the banana with a knife. We placed this piece of banana inside a cage (a modified Tomahawk trap with an additional inner door) located on one of the provisioning platforms (Supplementary Fig. S2a,b). However, we used this technique only with the first four individuals. Then, we decided to take advantage of particular situations in which a selected individual was ≥10 m away from other individuals and was being weighed on the balance or was visiting a provisioning platform (see above). On these occasions, we placed the banana piece with antiparasitic treatment on the platform or the balance as we were sure only the selected individual would have reached and eaten it. All these other attempts of drug administration were as successful as the others obtained using the cage, but had the advantage of being less stressful for the study subjects. The veterinarian E. V. supervised the drug administration as well as the subsequent monitoring of treated individuals following drug ingestion in order to document any adverse effect of the treatment, which we never observed.

### Experimental design

We used an experimental split-plot design that consisted of the following four treatment groups: (1) high food provisioning and antiparasitic treatment (F + A + ), (2) high food provisioning in the absence of antiparasitic treatment (F + A−), (3) low food provisioning and antiparasitic treatment (F−A+), and (4) control (i.e. individuals not treated with drugs in the group under low provisioning regime; F−A−) (Supplementary Fig. S3).

Following this scheme, during winter 2013 we supplied the Spot group with a high provisioning regime during 80 days, while the Macuco group was fed following a low provisioning regime during 66 days. Then, in winter 2014, we switched the two provisioning regimes between the two groups: the Spot group received a low provisioning during 29 days, while the Macuco group relied on a high provisioning during 60 days. In winter 2013, we administered antiparasitic drugs to three individuals (two females and one male) within the Spot group, and seven individuals (three males and four females) within the Macuco group. In winter 2014, we supplied the treatment to four individuals (two males and two females) in the Spot group, and five individuals in the Macuco group (two males and three females) (Supplementary Table S6 and Fig. S3). The selection of the individuals to be treated with antiparasitic drugs each year was stratified by sex and social rank.

All research reported in this article complied with the protocols approved by the Ethics Committee of the Argentine Society for the Study of Mammals^65^ and adhered to the legal requirements of Argentina. All research protocols were reviewed and approved by the National Parks Administration of Argentina.

### Data analysis

We described parasitic infections in terms of presence/absence of a given parasite, specific richness, occurrence of multiple infections and parasite load (eggs or larvae). Each individual’s faecal sample was classified as positive or negative for infection with a given parasite species. We also recorded richness (defined as the number of parasite species documented in each sample) and the frequency of infections by multiple species (i.e. the proportions of samples with > 1 parasite species), as multiple infections are associated with higher morbidity and mortality^46^. Finally, we also assessed parasite load, by estimating burdens of parasite eggs or larvae in faeces (eggs or larvae/sample)^17,66^. Parasite load is frequently reported to describe infections^17,66^, however because it is highly variable, even over the day, it may not be indicative of actual infection intensity, thus it should be considered with caution^46^. In our study, we used load to test the efficacy of the antiparasitic treatment in reducing parasite burden. We assumed that the action of antiparasitic drugs would have been completed around 7 days after application^67^. Thus, for data analysis we classified individuals as “Antiparasitic-Treated” from 7 days following the drug administration onwards.

In order to evaluate changes in the physical condition of an individual across treatments, we used repeated measures of body mass across the two winters. We averaged weight measures per day whenever an individual was weighted more than once during the same day. Then, we only used repeated measures taken on different days for each individual as point data for subsequent analyses.

We evaluated how provisioning and antiparasitic treatments affect parasitism with Generalised Linear Mixed Models (GLMM). These models included presence/absence of the most prevalent parasites, eggs or larval load (number of eggs or larvae/sample), parasite richness and presence of multiple infections as response variables, and a combination of one to three predictor variables: two treatments (provisioning and antiparasitic), and faecal sample weight as a potential confounder. Random effects included “individual ID” nested within “group ID”. We fitted the models with parasite presence or multiple infection probability as response variables to binomial error distributions. As for count data (i.e. number of eggs or larvae per sample; number of different parasite taxa per sample) we fit a Poisson error distribution when the data was not overdispersed (i. e. the dispersion parameter φ was about 1^68^), and used negative binomial distributions when there was overdispersion (φ > 1, for *Filariopsis* sp. and Hymenolepididae loads). As link functions, relating the linear predictor to the expected value of the response, we used log-link for Poisson and negative binomial distributions, and logit link for binomial error distributions^69^.

Similarly, we used GLMM to evaluate the effects of food provisioning, antiparasitic treatment, as well as sex as a control factor, on body mass in capuchin monkeys. “Individual ID” and “group ID” were the two nested random effects. We fitted these body mass models to normal error distributions^69^. We checked model assumptions (normality and homogeneity of residuals) and no significant violations or influential cases were detected. In all cases, we decided not to include the interaction terms (food provisioning x antiparasitic treatment), due to the small sample size and limited statistical power of our field experiment.

For GLMMs involving both parasite-related and body mass response variables we originally considered two to three (sex, social rank and female lactancy status) potentially confounding factors, which were included as fixed effects. Individual dominance status (high vs. low ranking; Supplementary Table S1) was assigned on the basis of a hierarchy generated by entering all observed agonistic interactions including aggressions, spatial displacements and submissive behaviours into a dominance matrix using Matman 1.1 (Noldus Information Technology 2003)^70^. However, due to our restricted sample size leading to issues of model convergence, after checking the lack of important effects of rank and lactancy status on the response variables, we decided not to include these covariates further in our modelling. Following the same logic, we opted not to include “year” as a further random effect in our models.

We used a second-order Akaike’s Information Criterion (AICc) to compare among competitive models. Models were ranked using AICc model weights. Since we ran an experiment with a small number of orthogonal factors, the use of full models is usually most appropriate (rather than considering more parsimonious models)^71^. Thus, we estimated effect sizes and measures of precision (95% confidence interval) for each response variable from the full model containing all predictor or control variables of interest. For models involving parasite load as response variable, we were not able to run models with multiple predictor variables due to a small sample size leading to a lack of model convergence. In these cases, we restricted our analyses to ranking univariate models (including one covariate at a time) according to AICc values. We performed all analyses with R software (v. 3.3.0; R Core Team 2016). In particular, we run GLMMs using R packages *nlme* ^72^ for models involving normal distribution of residuals, and *lme4* ^73^ for binomial, Poisson and negative binomial distributions. Model selection was performed using the R package *MuMIn* ^74^.

### Data availability

The authors declare that all data supporting the findings of this study are available within the paper and its Supplementary Information files.

## Electronic supplementary material

Supplementary Material

Dataset
