# Supplementary material for: Experimental testing of reciprocal effects of nutrition and parasitism in wild black capuchin monkeys
Source: Sci Rep. 2017 Oct 6;7:12778. doi: 10.1038/s41598-017-12803-8 (PMC5630591; doi:10.1038/s41598-017-12803-8)

## Supplementary material to the article:

"Experimental testing of reciprocal effects of nutrition and parasitism in wild black capuchin monkeys"

Ilaria Agostini* [1,2], Ezequiel Vanderhoeven [2,3], Mario S. Di Bitetti [1,2,4], Pablo M. Beldomenico [5]

[1] *Instituto de Biología Subtropical (IBS), Universidad Nacional de Misiones (UNaM), Consejo Nacional de Investigaciones Científicas y Técnicas (CONICET), Puerto Iguazú (Misiones), Argentina*

[2] *Asociación Civil Centro de Investigaciones del Bosque Atlántico (CeIBA), Puerto Iguazú, Argentina*

[3] *Instituto Nacional de Medicina Tropical (INMeT), Puerto Iguazú, Argentina*

[4] *Facultad de Ciencias Forestales, UNaM, Argentina*

[5] *Laboratorio de Ecología de Enfermedades, ICIVET LITORAL, UNL- CONICET, Esperanza (Santa Fe), Argentina*

Correspondence to:

Ilaria Agostini, Instituto de Biología Subtropical – sede Iguazú, CONICET-UNaM, Calle Bertoni 85, CP 3370 Puerto Iguazú, Misiones, Argentina. E-mail: agostini.ilaria@gmail.com

**Table S1. Summary of the study individuals' characteristics (age-sex class, social rank, treatments received, as well as number of repeated faecal samples and body weight (BW) measures obtained for each year. (a) MACUCO group; (b) SPOT group.**

**(a)**

| Year | Ind. ID | Age-sex | Rank | Provisioning | Antiparasitic | # Fecal samples | # BW measures |
|---|---|---|---|---|---|---|---|
| **2013** | CRD | ADF | Low | Low | Not Treated | 8 | 1 |
| | EST | ADF | High | Low | Treated | 10 | 1 |
| | ING | ADF | Low | Low | Treated | 9 | - |
| | MAW | ADF | Low | Low | Not Treated | 9 | - |
| | OFE | ADF | Low | Low | Treated | 13 | 3 |
| | SOL | ADF | Low | Low | Not Treated | 7 | - |
| | THE | ADF | High | Low | Treated | 14 | 5 |
| | YOL | ADF | Low | Low | Not Treated | 10 | - |
| | EDU | ADM | High | Low | Treated | 18 | 5 |
| | ERN | ADM | High | Low | Not Treated | 14 | 5 |
| | GAB | ADM | Low | Low | Not Treated | 0 | - |
| | MGO | ADM | Low | Low | Not Treated | 11 | - |
| | SRG | ADM | High | Low | Treated | 13 | 4 |
| | SRP | ADM | Low | Low | Not Treated | 9 | - |
| | TIT | ADM | Low | Low | Treated | 5 | - |
| | FEN | SUBF | Low | Low | Not Treated | 0 | - |
| | DAL | SUBM | Low | Low | Not Treated | 2 | - |
| | MAV | SUBM | Low | Low | Not Treated | 3 | - |
| | RIC | SUBM | Low | Low | Not Treated | 4 | - |
| **2014** | CRD | ADF | Low | High | Treated | 31 | 2 |
| | EST | ADF | High | High | Not Treated | 19 | 1 |
| | FEN | ADF | Low | High | Not Treated | 18 | - |
| | ING | ADF | Low | High | Not Treated | 25 | - |
| | MAW | ADF | Low | High | Treated | 21 | 2 |
| | OFE | ADF | Low | High | Not Treated | 20 | - |
| | SOL | ADF | Low | High | Treated | 17 | - |
| | THE | ADF | High | High | Not Treated | 16 | 1 |
| | EDU | ADM | High | High | Not Treated | 25 | 2 |
| | ERN | ADM | High | High | Treated | 30 | 4 |
| | SRG | ADM | High | High | Not Treated | 34 | 3 |
| | ESB | SUBF | Low | High | Not Treated | 0 | - |
| | MOR | SUBF | Low | High | Not Treated | 0 | - |
| | MAV | SUBM | Low | High | Not Treated | 12 | - |
| | RIC | SUBM | Low | High | Treated | 37 | - |

**(b)**

| Year | Ind. ID | Age-sex | Rank | Provisioning | Antiparasitic | # Fecal samples | # BW measures |
|---|---|---|---|---|---|---|---|
| 2013 | BIA | ADF | High | High | Not Treated | 10 | 5 |
| | DAN | ADF | Low | High | Not Treated | 8 | 3 |
| | EVA | ADF | Low | High | Treated | 14 | 2 |
| | JOS | ADF | Low | High | Treated | 12 | 2 |
| | RIN | ADM | High | High | Not Treated | 10 | - |
| | TET | ADM | High | High | Treated | 11 | - |
| | TRU | ADM | High | High | Not Treated | 7 | - |
| | FRA | SUBF | Low | High | Not Treated | 0 | - |
| 2014 | BIA | ADF | High | Low | Treated | 36 | 5 |
| | DAN | ADF | Low | Low | Treated | 9 | 1 |
| | EVA | ADF | Low | Low | Not Treated | 19 | 5 |
| | FRA | ADF | Low | Low | Not Treated | 1 | - |
| | JOS | ADF | Low | Low | Not Treated | 13 | - |
| | BOR | ADM | Low | Low | Not Treated | 10 | - |
| | HOR | ADM | Low | Low | Not Treated | 8 | - |
| | PAC | ADM | Low | Low | Not Treated | 2 | - |
| | RIN | ADM | High | Low | Treated | 17 | - |
| | TET | ADM | High | Low | Not Treated | 4 | - |
| | TRU | ADM | High | Low | Treated | 12 | - |
| | CAM | SUBM | Low | Low | Not Treated | 0 | - |
| | ROB | SUBM | Low | Low | Not Treated | 20 | - |

ADF = adult female; ADM = adult male; SUBF = subadult female; SUBM = subadult male.

**Table S2. Parasite taxa of both study groups with prevalence (percentage of individuals infected of the total number of sampled individuals) and burden range values (number of eggs or larvae found in faecal samples).**

| Parasite | Prevalence (%) | | Burden (range) | |
|---|---|---|---|---|
| | Winter 2013 | Winter 2014 | Winter 2013 | Winter 2014 |
| *Filariopsis* sp. | 54%* | 72%** | 1-34 (n = 30) | 1-21 (n = 37) |
| Hymenolepididae | 58%* | 64%** | 1-128 (n = 28) | 1-262 (n = 33) |
| *Strongyloides* sp. | 21%* | 48%** | 1-3 (n = 5) | 1-3 (n = 16) |
| *Ascaris* sp. | 8%* | 12%** | 1 (n = 2) | 1-2 (n = 3) |
| Spiruridae | 8%* | 4%** | 1 (n = 2) | 3 (n = 1) |
| *Trichuris* sp. | 8%* | 0%** | 2 (n = 2) | - (n = 0) |
| Subuluridae | 4%* | 4%** | 2 (n = 1) | 17 (n = 1) |
| Trematode | 4%* | 4%** | 1 (n = 1) | 1 (n = 1) |

* Total number of sampled individuals in 2013 = 24; ** total number of sampled individuals in 2014 = 25; n = number of faecal samples infected with a given parasite.

**Table S3. Mean ± SE and range of body weight (kg) for adult females and males of the two study groups during the two consecutive winters**. N = number of individuals weighted for each sex category.

| Field season | Sex | Body weight (mean ± SE) | Range | N |
|---|---|---|---|---|
| Winter 2013 | Females | 2.395 ± 0.096 | 1.930-2.710 | 8 |
| | Males | 3.227 ± 0.116 | 2.760-3.475 | 3 |
| Winter 2014 | Females | 2.644 ± 0.052 | 2.125-2.887 | 9 |
| | Males | 3.831 ± 0.159 | 3.463-4.100 | 4 |

**Table S4. Composition of the two study groups (Macuco and Spot) across the two winters 2013 and 2014.**

| Year | MACUCO group | | SPOT group | |
|---|---|---|---|---|
| | Sex-Age class | # of individuals | Sex-Age class | # of individuals |
| 2013 | ADM | 7 | ADM | 3 |
| | ADF | 8 | ADF | 4 |
| | SUBAD-M | 3 | SUBAD-M | 0 |
| | SUBAD-F | 1 | SUBAD-F | 1 |
| | JUV | 5 | JUV | 6 |
| | INF | 3 | INF | 3 |
| | **TOTAL** | **27** | **TOTAL** | **17** |
| 2014 | ADM | 3 | ADM | 5 |
| | ADF | 8 | ADF | 5 |
| | SUBAD-M | 1 | SUBAD-M | 2 |
| | SUBAD-F | 2 | SUBAD-F | 0 |
| | JUV | 5 | JUV | 7 |
| | INF | 4 | INF | 2 |
| | **TOTAL** | **23** | **TOTAL** | **21** |

ADM = adult males; ADF = adult females; SUBAD-M = subadult males; SUBAD-F = subadult females, JUV = juveniles; INF = infants.

**Table S5. Fixed factors from Generalized Linear Mixed Models explaining variation in (a) *Filariopsis* sp. larval load, and (b) Hymenolepididae egg load.** Comparison of AICc values of univariate models. For all models the only random effect considered was Group. Explanatory variables are listed in order of increasing AICc. See methods for details.

| Response variable | Predictor and control factors | K | AICc |
|---|---|---|---|
| **(a) *Filariopsis* sp. load** | Antiparasitic | 4 | 310.617 |
| | Null model | 3 | 313.283 |
| | Provisioning | 4 | 314.363 |
| | Fecal sample weight | 4 | 314.871 |
| **(b) Hymenolepididae load** | Fecal sample weight | 4 | 444.028 |
| | Antiparasitic | 4 | 448.711 |
| | Null model | 3 | 449.372 |
| | Provisioning | 4 | 450.499 |

**Generalised Linear Mixed Models full outputs for Table S4. Comparison of AICc values of univariate models for (a) *Filariopsis* sp. load, and (b) Hymenolepididae load.**

Response variables: FilarLOAD, HymenolLOAD. Fixed effects= Antiparasitic [levels = AntiparasiticNT (Not Treated), AntiparasiticT (Treated)], Provisioning [levels = ProvisioningLOW (low regime), ProvisioningHIGH (high regime)], FecalSampleW (Fecal Sample's Weight). Random effect = Group.

(a) ***Filariopsis* sp. load**

```
# Using negative binomial distribution, lme4 package. Due to convergence issu
es, we run only univariate models and then compare AIC values.Due to the same
convergence issues, we retained only ggroup (not individuals) as a random eff
ect in the models.


> BN1 <- glmer.nb(FilarLOAD ~ Antiparasitic + (1|Group), data = Filar)
> summary(BN1)

Generalized linear mixed model fit by maximum likelihood
  (Laplace Approximation) [glmerMod]
 Family: Negative Binomial(1.3038)  ( log )
Formula: FilarLOAD ~ Antiparasitic + (1 | Group)
   Data: Filar

     AIC      BIC   logLik deviance df.resid
   310.6    319.4   -151.3    302.6       63

Scaled residuals:
    Min      1Q  Median      3Q     Max
-0.7020 -0.7020 -0.4273  0.1221  8.3631

Random effects:
 Groups Name        Variance Std.Dev.
 Group  (Intercept) 0        0
Number of obs: 67, groups:  Group, 2

Fixed effects:
              Estimate Std. Error z value Pr(>|z|)
```

```
(Intercept)     0.4796     0.3265   1.469   0.1419
AntiparasiticNT   0.7889     0.3550   2.222   0.0263 *
---
Signif. codes:  0 ‘***’ 0.001 ‘**’ 0.01 ‘*’ 0.05 ‘.’ 0.1 ‘ ’ 1

Correlation of Fixed Effects:
            (Intr)
AntiparasiticNT -0.920

> BN2 <- glmer.nb(FilarLOAD ~ Provisioning + (1|Group), data = Filar)
> summary(BN2)

Generalized linear mixed model fit by maximum likelihood
  (Laplace Approximation) [glmerMod]
 Family: Negative Binomial(1.2324)  ( log )
Formula: FilarLOAD ~ Provisioning + (1 | Group)
   Data: Filar

     AIC      BIC   logLik deviance df.resid
   314.4    323.2   -153.2    306.4       63

Scaled residuals:
    Min      1Q  Median      3Q     Max
-0.6914 -0.5947 -0.5947 -0.1583 10.3078

Random effects:
 Groups Name        Variance Std.Dev.
 Group  (Intercept) 0        0
Number of obs: 67, groups:  Group, 2

Fixed effects:
             Estimate Std. Error z value Pr(>|z|)
(Intercept)    1.2792     0.1845   6.932 4.15e-12 ***
ProvisioningLOW  -0.2496     0.2597  -0.961    0.337
---
Signif. codes:  0 ‘***’ 0.001 ‘**’ 0.01 ‘*’ 0.05 ‘.’ 0.1 ‘ ’ 1

Correlation of Fixed Effects:
            (Intr)
ProvisioningLOW -0.711


> BN3 <- glmer.nb(FilarLOAD ~ FecalSampleW  + (1|Group), data = Filar)
> summary(BN3)
Generalized linear mixed model fit by maximum likelihood
  (Laplace Approximation) [glmerMod]
 Family: Negative Binomial(1.2224)  ( log )
Formula: FilarLOAD ~ FecalSampleW + (1 | Group)
   Data: Filar

     AIC      BIC   logLik deviance df.resid
   314.9    323.7   -153.4    306.9       63

Scaled residuals:
    Min      1Q  Median      3Q     Max
-0.7815 -0.6327 -0.6327 -0.0264  9.3700

Random effects:
 Groups Name        Variance Std.Dev.
 Group  (Intercept) 9.59e-14 3.097e-07
Number of obs: 67, groups:  Group, 2

Fixed effects:
            Estimate Std. Error z value Pr(>|z|)
(Intercept)   2.0103     1.3660   1.472    0.141
FecalSampleW  -0.2943     0.4679  -0.629    0.529

Correlation of Fixed Effects:
            (Intr)
FecalSampleW -0.995
```

```
>

> BN0 <- glmer.nb(FilarLOAD ~ 1 + (1|Group), data = Filar)
> summary(BN0)

Generalized linear mixed model fit by maximum likelihood
  (Laplace Approximation) [glmerMod]
 Family: Negative Binomial(1.2143)  ( log )
Formula: FilarLOAD ~ 1 + (1 | Group)
   Data: Filar

     AIC      BIC   logLik deviance df.resid
   313.3    319.9   -153.6    307.3       64

Scaled residuals:
    Min      1Q  Median      3Q     Max
-0.6425 -0.6425 -0.6425 -0.0528  9.0877

Random effects:
 Groups Name        Variance Std.Dev.
 Group  (Intercept) 0        0
Number of obs: 67, groups:  Group, 2

Fixed effects:
            Estimate Std. Error z value Pr(>|z|)
(Intercept)   1.1566     0.1303   8.874   <2e-16 ***
---
Signif. codes:  0 '***' 0.001 '**' 0.01 '*' 0.05 '.' 0.1 ' ' 1


> # Comparing models with Akaike Information Criterion (AIC).

> AIC(BN1, BN2, BN3, BN0)
    df      AIC
BN1  4 310.6171
BN2  4 314.3627
BN3  4 314.8713
BN0  3 313.2827

> # The best model contains Antiparasitic treatment as a fixed factor and is
> 2 AIC from the other three models.
```

**(b) Hymenolepididae load**

```
# Using negative binomial distribution, lme4 package. Due to convergence issu
es, we run only univariate models and then compare AIC values.Due to the same
convergence issues, we retained only ggroup (not individuals) as a random eff
ect in the models.

> BN1 <- glmer.nb(HymenolLOAD ~ Provisioning + (1|Group), data = Hymenol)
> summary(BN1)
Generalized linear mixed model fit by maximum likelihood
  (Laplace Approximation) [glmerMod]
 Family: Negative Binomial(0.571)  ( log )
Formula: HymenolLOAD ~ Provisioning + (1 | Group)
   Data: Hymenol

     AIC      BIC   logLik deviance df.resid
   450.5    458.9   -221.2    442.5       57

Scaled residuals:
    Min      1Q  Median      3Q     Max
-0.7236 -0.6364 -0.5243 -0.1968  5.7787

Random effects:
 Groups Name        Variance Std.Dev.
 Group  (Intercept) 0.507    0.7121
```

```
Number of obs: 61, groups:  Group, 2

Fixed effects:
                 Estimate Std. Error z value Pr(>|z|)    
(Intercept)        2.3767     0.5796   4.101 4.12e-05 ***
ProvisioningLOW    0.3527     0.3715   0.949    0.342    
---
Signif. codes:  0 ‘***’ 0.001 ‘**’ 0.01 ‘*’ 0.05 ‘.’ 0.1 ‘ ’ 1

Correlation of Fixed Effects:
            (Intr)
ProvisioningLOW -0.391
> 
> BN2 <- glmer.nb(HymenolLOAD ~ Antiparasitic + (1|Group), data = Hymenol)
> summary(BN2)
Generalized linear mixed model fit by maximum likelihood
  (Laplace Approximation) [glmerMod]
 Family: Negative Binomial(0.5855)  ( log )
Formula: HymenolLOAD ~ Antiparasitic + (1 | Group)
   Data: Hymenol

     AIC      BIC   logLik deviance df.resid 
   448.7    457.2   -220.4    440.7       57 

Scaled residuals: 
    Min      1Q  Median      3Q     Max 
-0.7115 -0.6360 -0.5357 -0.1342  5.3800 

Random effects:
 Groups Name        Variance Std.Dev.
 Group  (Intercept) 0.6066   0.7788  
Number of obs: 61, groups:  Group, 2

Fixed effects:
                Estimate Std. Error z value Pr(>|z|)   
(Intercept)       2.0316     0.6554   3.100  0.00194 **
AntiparasiticNT   0.7020     0.4042   1.737  0.08240 . 
---
Signif. codes:  0 ‘***’ 0.001 ‘**’ 0.01 ‘*’ 0.05 ‘.’ 0.1 ‘ ’ 1

Correlation of Fixed Effects:
            (Intr)
AntiparasiticNT -0.473
> 
> BN3 <- glmer.nb(HymenolLOAD ~ PesoMuestra + (1|Group), data = Hymenol)
> summary(BN3)
Generalized linear mixed model fit by maximum likelihood
  (Laplace Approximation) [glmerMod]
 Family: Negative Binomial(0.6193)  ( log )
Formula: HymenolLOAD ~ FecalSampleW + (1 | Group)
   Data: Hymenol

     AIC      BIC   logLik deviance df.resid 
   444.0    452.5   -218.0    436.0       57 

Scaled residuals: 
    Min      1Q  Median      3Q     Max 
-0.7533 -0.6611 -0.4679 -0.1221  5.9721 

Random effects:
 Groups Name        Variance Std.Dev.
 Group  (Intercept) 0.5077   0.7126  
Number of obs: 61, groups:  Group, 2

Fixed effects:
             Estimate Std. Error z value Pr(>|z|)   
(Intercept)   -0.9654     1.2328  -0.783  0.43358   
FecalSampleW   1.2327     0.3922   3.143  0.00167 **
---
Signif. codes:  0 ‘***’ 0.001 ‘**’ 0.01 ‘*’ 0.05 ‘.’ 0.1 ‘ ’ 1
```

```
Correlation of Fixed Effects:
            (Intr)
FecalSampleW -0.902
>
> BN0 <- glmer.nb(HymenolLOAD ~ 1 + (1|Group), data = Hymenol)
> summary(BN0)
Generalized linear mixed model fit by maximum likelihood
  (Laplace Approximation) [glmerMod]
 Family: Negative Binomial(0.5657)  ( log )
Formula: HymenolLOAD ~ 1 + (1 | Group)
   Data: Hymenol

     AIC      BIC   logLik deviance df.resid
   449.4    455.7   -221.7    443.4       58

Scaled residuals:
    Min      1Q  Median      3Q     Max
-0.7177 -0.6129 -0.5038 -0.2857  6.3011

Random effects:
 Groups Name        Variance Std.Dev.
 Group  (Intercept) 0.5685   0.754
Number of obs: 61, groups:  Group, 2

Fixed effects:
            Estimate Std. Error z value Pr(>|z|)
(Intercept)   2.6041     0.5616   4.637 3.54e-06 ***
---
Signif. codes:  0 '***' 0.001 '**' 0.01 '*' 0.05 '.' 0.1 ' ' 1
>
> AIC(BN1, BN2, BN3, BN0)
    df      AIC
BN1  4 450.4992
BN2  4 448.7114
BN3  4 444.0278
BN0  3 449.3724
> # BN3 is the best model with Fecal Sample Weight as a fixed factor, and the
second-ranked contains the antiparasitic treatment but is > 2 AIC from the be
st model.
```

**GLMM full outputs for Tables 1 and 2. Model selection and full model summary for (a) *Filariopsis* sp. infection, (b) Hymenolepididae infection, (c) parasite richness, and (d) probability of Multiple Infections.**

Response variables: presFilar (probability of infection with *Filariopsis*), presHymenol (probability of infection with Hymenolepididae), Richness (parasite richness), MultipleInfec (probability of multiple infections). Fixed effects= Antiparasitic [levels = AntiparasiticNT (Not Treated), AntiparasiticT (Treated)], Provisioning [levels = ProvisioningLOW (low regime), ProvisioningHIGH (high regime)], FecalSampleW (Fecal Sample's Weight). Random effects = Group, ID. Ind (individuals' identities).

### (a) *Filariopsis* sp. infection.

```
> ##### GLMM to see the influence of different factors on the presence of Fil
ariopsis in individuals. Since it is a 1-0 data set, we used a binomial distr
ibution. lme4 and MuMIn packages. We selected models and ranked them accordin
g to AICc weight.

options(na.action = "na.fail")

> mod.global <- glmer(presFilar ~ Provisioning + Antiparasitic +
```

```
FecalSampleW + (1|Group/ID.Ind), data = Filariopsis, family = bin
omial)
> 
> mod1 <- glmer(presFilar ~ Antiparasitic + FecalSampleW + (1|Gro
up/ID.Ind), data = Filariopsis, family = binomial)
> 
> mod2 <- glmer(presFilar ~ Provisioning + Antiparasitic +  (1|Gr
oup/ID.Ind), data = Filariopsis, family = binomial)
> 
> mod3 <- glmer(presFilar ~ Provisioning + FecalSampleW + (1|Grou
p/ID.Ind), data = Filariopsis, family = binomial)
> 
> mod4 <- glmer(presFilar ~ FecalSampleW + (1|Group/ID.Ind), data
= Filariopsis, family = binomial)
> 
> mod5 <- glmer(presFilar ~ Antiparasitic + (1|Group/ID.Ind), dat
a = Filariopsis, family = binomial)
> 
> mod6 <- glmer(presFilar ~ Provisioning + (1|Group/ID.Ind), data
= Filariopsis, family = binomial)
Warning message:
In checkConv(attr(opt, "derivs"), opt$par, ctrl = control$checkCo
nv,  :
  Model failed to converge with max|grad| = 0.0411894 (tol = 0.00
1, component 1)
> # mod6 does not converge.
> 
> modNull <- glmer(presFilar ~ 1 + (1|Group/ID.Ind), data = Filar
iopsis, family = binomial)
> summary(modNull)

Generalized linear mixed model fit by maximum likelihood
  (Laplace Approximation) [glmerMod]
 Family: binomial  ( logit )
Formula: presFilar ~ 1 + (1 | Group/ID.Ind)
   Data: Filariopsis

     AIC      BIC   logLik deviance df.resid
   431.6    445.2   -212.8    425.6      684

Scaled residuals:
    Min      1Q  Median      3Q     Max
-0.5115 -0.3844 -0.2637 -0.2122  4.7124

Random effects:
 Groups       Name        Variance  Std.Dev.
 ID.Ind:Group (Intercept) 6.576e-01 8.109e-01
 Group        (Intercept) 1.897e-10 1.377e-05
Number of obs: 687, groups:  ID.Ind:Group, 30; Group, 2

Fixed effects:
            Estimate Std. Error z value Pr(>|z|)
(Intercept)  -2.5113     0.2432  -10.33   <2e-16 ***
---
Signif. codes:  0 ‘***’ 0.001 ‘**’ 0.01 ‘*’ 0.05 ‘.’ 0.1 ‘ ’ 1

> # Model selection:

> MMI.1<-model.sel(mod.global, mod1, mod2, mod3, mod4, mod5, modN
ull, rank="AICc")
> MMI.1
Model selection table
           (Intrc)  PsMst PROVI Trtph df   logLik  AICc delta
mod.global  -5.894 0.8896     +     +  6 -206.703 425.5  0.00
mod3        -5.277 0.8601     +        5 -208.278 426.6  1.11
mod1        -5.417 0.8806           +  5 -208.644 427.4  1.85
mod4        -4.974 0.8590              4 -209.727 427.5  1.98
mod2        -3.312              +     +  5 -209.907 429.9  4.37
modNull     -2.511                     3 -212.781 431.6  6.07
mod5        -2.858                  +  4 -211.859 431.8  6.25
```

```
             weight
mod.global  0.393
mod3        0.225
mod1        0.156
mod4        0.146
mod2        0.044
modNull     0.019
mod5        0.017
Models ranked by AICc(x)
Random terms (all models):
‘1 | Group/ID.Ind’

> # select models 95% cumulative weight criteria
> output <- subset(MMI.1, cumsum(MMI.1$weight) <= .95)
> output
Model selection table
             (Intrc)  PsMst PROVI Trtph df   logLik  AICc delta
mod.global  -5.894 0.8896     +     +  6 -206.703 425.5  0.00
mod3        -5.277 0.8601     +        5 -208.278 426.6  1.11
mod1        -5.417 0.8806           +  5 -208.644 427.4  1.85
mod4        -4.974 0.8590              4 -209.727 427.5  1.98
             weight
mod.global  0.427
mod3        0.245
mod1        0.170
mod4        0.158
Models ranked by AICc(x)
Random terms (all models):
‘1 | Group/ID.Ind’

> # Full model with provisioning + antiparasitic treatment + feca
l sample weight, as explanatory variables.
>

summary(mod.global)
> summary(mod.global)
Generalized linear mixed model fit by maximum likelihood
  (Laplace Approximation) [glmerMod]
 Family: binomial  ( logit )
Formula:
presFilar ~ Provisioning + Antiparasitic + FecalSampleW + (1 | Gr
oup/ID.Ind)
Data: Filariopsis

     AIC      BIC   logLik deviance df.resid
   425.4    452.6   -206.7    413.4      681

Scaled residuals:
    Min      1Q  Median      3Q     Max
-0.7078 -0.3402 -0.2708 -0.1741  5.8401

Random effects:
 Groups       Name        Variance Std.Dev.
 ID.Ind:Group (Intercept) 0.75912  0.8713
 Group        (Intercept) 0.03314  0.1821
Number of obs: 687, groups:  ID.Ind:Group, 30; Group, 2

Fixed effects:
                  Estimate   Std. Error z value Pr(>|z|)
(Intercept)        -5.8939     1.3172  -4.475 7.66e-06 ***
ProvisioningLOW     0.5965     0.3061   1.949   0.0513 .
AntiparasiticNT     0.5946     0.3476   1.711   0.0872 .
FecalSampleW        0.8896     0.4141   2.148   0.0317 *
---
Signif. codes:  0 ‘***’ 0.001 ‘**’ 0.01 ‘*’ 0.05 ‘.’ 0.1 ‘ ’ 1

Correlation of Fixed Effects:
                 (Intr) Provis Antpar
ProvisioningLOW -0.216
AntiparasiticNT -0.287  0.169
```

```
FecalSampleW    -0.929  0.018  0.047

## estimating profile confidence intervals:
> confint(mod.global)

Computing profile confidence intervals ...
                     2.5 %      97.5 %
.sig01           0.432973005  1.471527
.sig02           0.000000000  1.694953
(Intercept)     -8.874284722 -3.602105
ProvisioningLOW  0.003035329  1.197668
AntiparasiticNT -0.059833848  1.311857
FecalSampleW     0.177686310  1.839702
```

### (b) Hymenolepididae infection

```
> mod.global <- glmer(presHymenol ~ Provisioning + Antiparasitic
+ FecalSampleW + (1|Group/ID.Ind), data = Hymenol, family = binom
ial)
>
> mod1 <- glmer(presHymenol ~ Antiparasitic + FecalSampleW + (1|G
roup/ID.Ind), data = Hymenol, family = binomial)
>
> mod2 <- glmer(presHymenol ~ Provisioning + Antiparasitic + (1|G
roup/ID.Ind), data = Hymenol, family = binomial)
>
> mod3 <- glmer(presHymenol ~ Provisioning + FecalSampleW + (1|Gr
oup/ID.Ind), data = Hymenol, family = binomial)
>
> mod4 <- glmer(presHymenol ~ FecalSampleW + (1|Group/ID.Ind), da
ta = Hymenol, family = binomial)
>
> mod5 <- glmer(presHymenol ~ Antiparasitic + (1|Group/ID.Ind), d
ata = Hymenol, family = binomial)
>
> mod6 <- glmer(presHymenol ~ Provisioning + (1|Group/ID.Ind), da
ta = Hymenol, family = binomial)
>
> modNull <- glmer(presHymenol ~ 1 + (1|Group/ID.Ind), data = Hym
enol, family = binomial)
> summary(modNull)
Generalized linear mixed model fit by maximum likelihood
  (Laplace Approximation) [glmerMod]
 Family: binomial  ( logit )
Formula: presHymenol ~ 1 + (1 | Group/ID.Ind)
   Data: Hymenol

     AIC      BIC   logLik deviance df.resid
   406.4    420.0   -200.2    400.4      684

Scaled residuals:
    Min      1Q  Median      3Q     Max
-0.6884 -0.3600 -0.2692 -0.2196  4.6195

Random effects:
 Groups       Name        Variance Std.Dev.
 ID.Ind:Group (Intercept) 0.5135   0.7166
 Group        (Intercept) 0.1771   0.4208
Number of obs: 687, groups:  ID.Ind:Group, 31; Group, 2

Fixed effects:
            Estimate Std. Error z value Pr(>|z|)
(Intercept)  -2.4097     0.3674  -6.558 5.44e-11 ***
---
Signif. codes:  0 ‘***’ 0.001 ‘**’ 0.01 ‘*’ 0.05 ‘.’ 0.1 ‘ ’ 1
```

```
> MMI.1<-model.sel(mod.global, mod1, mod2, mod3, mod4, mod5, mod6, modNull, r
ank="AICc")
> MMI.1
Model selection table
            (Intrc)   FSamW  Provi Antpar df   logLik  AICc delta
mod6         -2.717              +          4 -198.670 405.4  0.00
modNull      -2.410                         3 -200.196 406.4  1.03
mod2         -2.889              +      +   5 -198.478 407.0  1.65
mod3         -2.811 0.03427      +          5 -198.663 407.4  2.02
mod5         -2.495                     +   4 -200.135 408.3  2.93
mod4         -2.563 0.05528                 4 -200.177 408.4  3.01
mod.global   -3.017 0.04573      +      +   6 -198.466 409.1  3.66
mod1         -2.672 0.06239             +   5 -200.112 410.3  4.91
            weight
mod6         0.322
modNull      0.193
mod2         0.142
mod3         0.118
mod5         0.074
mod4         0.071
mod.global   0.052
mod1         0.028
Models ranked by AICc(x)
Random terms (all models):
‘1 | Group/ID.Ind’

> # select models 95% cumulative weight criteria
> output <- subset(MMI.1, cumsum(MMI.1$weight) <= .95)
> output
Model selection table
         (Intrc)   FSamW  Provi Antpar df   logLik  AICc delta
mod6      -2.717              +          4 -198.670 405.4  0.00
modNull   -2.410                         3 -200.196 406.4  1.03
mod2      -2.889              +      +   5 -198.478 407.0  1.65
mod3      -2.811 0.03427      +          5 -198.663 407.4  2.02
mod5      -2.495                     +   4 -200.135 408.3  2.93
mod4      -2.563 0.05528                 4 -200.177 408.4  3.01
         weight
mod6      0.350
modNull   0.209
mod2      0.154
mod3      0.128
mod5      0.081
mod4      0.078
Models ranked by AICc(x)
Random terms (all models):
‘1 | Group/ID.Ind’

> # > # Full model with provisioning + antiparasitic treatment + fecal sample
weight, as explanatory variables.
>
> summary(mod.global)
Generalized linear mixed model fit by maximum likelihood
  (Laplace Approximation) [glmerMod]
 Family: binomial  ( logit )
Formula:
presHymenol ~ Provisioning + Antiparasitic + FecalSampleW + (1 |
Group/ID.Ind)
   Data: Hymenol

     AIC      BIC   logLik deviance df.resid
   408.9    436.1   -198.5    396.9      681

Scaled residuals:
    Min      1Q  Median      3Q     Max
-0.6940 -0.3306 -0.2710 -0.2087  4.9950

Random effects:
 Groups       Name        Variance Std.Dev.
 ID.Ind:Group (Intercept) 0.45513  0.6746
```

```
 Group        (Intercept) 0.09093  0.3015
Number of obs: 687, groups:  ID.Ind:Group, 31; Group, 2

Fixed effects:
                  Estimate Std. Error z value Pr(>|z|)
(Intercept)       -3.01725    0.91569  -3.295 0.000984 ***
ProvisioningLOW    0.57185    0.30765   1.859 0.063058 .
AntiparasiticNT    0.20688    0.32383   0.639 0.522916
FecalSampleW       0.04573    0.28249   0.162 0.871408
---
Signif. codes:  0 ‘***’ 0.001 ‘**’ 0.01 ‘*’ 0.05 ‘.’ 0.1 ‘ ’ 1

Correlation of Fixed Effects:
             (Intr) Provisioning AntiparasiticNT
ProvisionLOW -0.224
AntiparasNT  -0.356     0.142
FecSampleW   -0.870    -0.028          0.061
## estimating profile confidence intervals:

> confint(mod.global)
Computing profile confidence intervals ...


                       2.5 %     97.5 %
.sig01            0.20115706  1.2370891
.sig02            0.00000000  1.7290528
(Intercept)      -5.02996959 -1.2658581
ProvisioningLOW  -0.04584869  1.2035181
AntiparasiticNT  -0.42539574  0.8876444
FecalSampleW     -0.48443622  0.6715771
```

**(c) Parasite richness**

```
> mod.global <- glmer(Richness ~ Antiparasitic + Provisioning + FecalSampleW
+ (1|Group/ID.Ind), data = ParRichness, family = poisson)
>
> mod1 <- glmer(Richness ~ Antiparasitic + FecalSampleW+ (1|Group/ID.Ind), da
ta = ParRichness, family = poisson) # no converge
Warning message:
In checkConv(attr(opt, "derivs"), opt$par, ctrl = control$checkConv,  :
  Model failed to converge with max|grad| = 0.00148275 (tol = 0.001, componen
t 1)
>
> mod2 <- glmer(Richness ~ Provisioning + Antiparasitic +  (1|Group/ID.Ind),
data = ParRichness, family = poisson)
>
> mod3 <- glmer(Richness ~ Provisioning + FecalSampleW+  (1|Group/ID.Ind), da
ta = ParRichness, family = poisson)  # no converge.
Warning message:
In checkConv(attr(opt, "derivs"), opt$par, ctrl = control$checkConv,  :
  Model failed to converge with max|grad| = 0.00206432 (tol = 0.001, componen
t 1)
>
> mod4 <- glmer(Richness ~ FecalSampleW+ (1|Group/ID.Ind), data = ParRichness
, family = poisson)
>
> mod5 <- glmer(Richness ~ Antiparasitic + (1|Group/ID.Ind), data = ParRichne
ss, family = poisson)
>
> mod6 <- glmer(Richness ~ Provisioning + (1|Group/ID.Ind), data = ParRichnes
s, family = poisson)
>
> modNull <- glmer(Richness ~ 1 + (1|Group/ID.Ind), data = ParRichness, famil
y = poisson)
> summary(modNull)
Generalized linear mixed model fit by maximum likelihood
  (Laplace Approximation) [glmerMod]
```

```
 Family: poisson  ( log )
Formula: Richness ~ 1 + (1 | Group/ID.Ind)
   Data: ParRichness

     AIC      BIC   logLik deviance df.resid
   887.0    900.6   -440.5    881.0      684

Scaled residuals:
    Min      1Q  Median      3Q     Max
-0.5641 -0.5238 -0.5040 -0.4685  5.1779

Random effects:
 Groups       Name        Variance Std.Dev.
 ID.Ind:Group (Intercept) 0.03144  0.1773
 Group        (Intercept) 0.00000  0.0000
Number of obs: 687, groups:  ID.Ind:Group, 31; Group, 2

Fixed effects:
            Estimate Std. Error z value Pr(>|z|)
(Intercept) -1.32785    0.08319  -15.96   <2e-16 ***
---
Signif. codes:  0 '***' 0.001 '**' 0.01 '*' 0.05 '.' 0.1 ' ' 1

> MMI.1<-model.sel(mod.global,mod2, mod4, mod5, mod6, modNull, rank="AICc")
> MMI.1
Model selection table
           (Intrc)  FSamW  Provi Antpa df   logLik  AICc delta
mod.global  -2.562 0.2783     +     +  6 -433.803 879.7  0.00
mod2        -1.771            +     +  5 -435.218 880.5  0.79
mod6        -1.547            +        4 -436.498 881.1  1.32
mod4        -2.119 0.2805              4 -439.050 886.2  6.43
modNull     -1.328                     3 -440.502 887.0  7.31
mod5        -1.501                  +  4 -439.654 887.4  7.64
           weight
mod.global  0.439
mod2        0.295
mod6        0.227
mod4        0.018
modNull     0.011
mod5        0.010
Models ranked by AICc(x)
Random terms (all models):
'1 | Group/ID.Ind'

> output <- subset(MMI.1, cumsum(MMI.1$weight) <= .95)
> output
Model selection table
           (Intrc)  FSamW  Provi Antpa df   logLik  AICc delta
mod.global  -2.562 0.2783     +     +   6 -433.803 879.7  0.00
mod2        -1.771            +     +   5 -435.218 880.5  0.79
           weight
mod.global  0.598
mod2        0.402
Models ranked by AICc(x)
Random terms (all models):
'1 | Group/ID.Ind'

### effect size on the basis of full model (mod.global)
summary(mod.global)

Generalized linear mixed model fit by maximum likelihood
  (Laplace Approximation) [glmerMod]
 Family: poisson  ( log )
Formula:
Richness ~ Antiparasitic + Provisioning + FecalSampleW + (1 | Gro
up/ID.Ind)
   Data: ParRichness

     AIC      BIC   logLik deviance df.resid
   879.6    906.8   -433.8    867.6      681
```

```
Scaled residuals:
    Min      1Q  Median      3Q     Max 
-0.6437 -0.5255 -0.4747 -0.3551  5.0573 

Random effects:
 Groups       Name        Variance Std.Dev.
 ID.Ind:Group (Intercept) 0.02113  0.1454  
 Group        (Intercept) 0.00000  0.0000  
Number of obs: 687, groups:  ID.Ind:Group, 31; Group, 2

Fixed effects:
                Estimate Std. Error z value Pr(>|z|)    
(Intercept)      -2.5620     0.5390  -4.753    2e-06 ***
AntiparasiticNT   0.2902     0.1795   1.617   0.1058    
ProvisioningLOW   0.4452     0.1525   2.920   0.0035 ** 
FecalSampleW      0.2783     0.1757   1.584   0.1132    
---
Signif. codes:  0 ‘***’ 0.001 ‘**’ 0.01 ‘*’ 0.05 ‘.’ 0.1 ‘ ’ 1

Correlation of Fixed Effects:
                (Intr) AntiparasiticNT  ProvisioningLOW
AntiparasiticNT  -0.304                                
ProvisioningLOW  -0.175     0.096                      
FecalSampleW     -0.935     0.031             -0.014   

## estimating profile confidence intervals:
confint(mod.global)
Computing profile confidence intervals ...

                      2.5 %      97.5 %
.sig01           0.00000000  0.4056204
.sig02           0.00000000  0.2896494
(Intercept)     -3.70082160 -1.5755947
AntiparasiticNT -0.05164119  0.6544597
ProvisioningLOW  0.14822840  0.7481103
FecalSampleW    -0.04288037  0.6502666
```

### (d) Probability of Multiple Infections

```
> Mix.global <- glmer(MultipleInfec ~ Provisioning + Antiparasitic + FecalSam
pleW + (1|Group/ID.Ind), data = MultINF, family = binomial)
>
> Mix1 <- glmer(MultipleInfec ~ Provisioning + Antiparasitic + (1|Group/ID.In
d), data = MultINF, family = binomial)
>
> Mix2 <- glmer(MultipleInfec ~ Provisioning + FecalSampleW + (1|Group/ID.Ind
), data = MultINF, family = binomial)
>
> Mix3 <- glmer(MultipleInfec ~ Antiparasitic + FecalSampleW + (1|Group/ID.In
d), data = MultINF, family = binomial)   # no converge.
Warning message:
In checkConv(attr(opt, "derivs"), opt$par, ctrl = control$checkConv,  :
  Model failed to converge with max|grad| = 0.00984842 (tol = 0.001, componen
t 1)
>
> Mix4 <- glmer(MultipleInfec ~ Antiparasitic + (1|Group/ID.Ind), data = Mult
INF, family = binomial)
>
> Mix5 <- glmer(MultipleInfec ~ FecalSampleW + (1|Group/ID.Ind), data = MultI
NF, family = binomial)
>
> Mix6 <- glmer(MultipleInfec ~ Provisioning + (1|Group/ID.Ind), data = MultI
NF, family = binomial)
>
```

```
> MixNull <- glmer(MultipleInfec ~ 1 + (1|Group/ID.Ind), data = MultINF, fami
ly = binomial)
> summary(MixNull)
Generalized linear mixed model fit by maximum likelihood
  (Laplace Approximation) [glmerMod]
 Family: binomial  ( logit )
Formula: MultipleInfec ~ 1 + (1 | Group/ID.Ind)
   Data: MultINF

     AIC      BIC   logLik deviance df.resid
   193.8    207.4    -93.9    187.8      684

Scaled residuals:
    Min      1Q  Median      3Q     Max
-0.2136 -0.1749 -0.1694 -0.1655  5.9269

Random effects:
 Groups       Name        Variance Std.Dev.
 ID.Ind:Group (Intercept) 0.1647   0.4058
 Group        (Intercept) 0.0000   0.0000
Number of obs: 687, groups:  ID.Ind:Group, 31; Group, 2

Fixed effects:
            Estimate Std. Error z value Pr(>|z|)
(Intercept)   -3.510      0.305  -11.51   <2e-16 ***
---
Signif. codes:  0 '***' 0.001 '**' 0.01 '*' 0.05 '.' 0.1 ' ' 1

> # model selection.
> library(MuMIn)
> MMI.1<-model.sel(Mix.global,Mix1, Mix2, Mix4, Mix5, Mix6, MixNull, rank="AI
Cc")
> MMI.1
Model selection table
           (Intrc)    FSamW  Provi Antpa df  logLik  AICc delta
Mix6        -4.114               +         4 -90.986 190.0  0.00
Mix1        -4.568               +     +   5 -90.442 191.0  0.94
Mix2        -3.572 -0.1978       +         5 -90.876 191.8  1.81
Mix.global  -4.070 -0.1787       +     +   6 -90.352 192.8  2.80
MixNull     -3.509                         3 -93.888 193.8  3.78
Mix4        -3.842                     +   4 -93.551 195.2  5.13
Mix5        -3.106 -0.1439                 4 -93.827 195.7  5.68
           weight
Mix6        0.390
Mix1        0.244
Mix2        0.158
Mix.global  0.096
MixNull     0.059
Mix4        0.030
Mix5        0.023
Models ranked by AICc(x)
Random terms (all models):
'1 | Group/ID.Ind'


> # select models 95% cumulative weight criteria
> output <- subset(MMI.1, cumsum(MMI.1$weight) <= .95)
> output
Model selection table
           (Intrc)    FSamW  Provi Antpa df  logLik  AICc delta
Mix6        -4.114               +         4 -90.986 190.0  0.00
Mix1        -4.568               +     +   5 -90.442 191.0  0.94
Mix2        -3.572 -0.1978       +         5 -90.876 191.8  1.81
Mix.global  -4.070 -0.1787       +     +   6 -90.352 192.8  2.80
MixNull     -3.509                         3 -93.888 193.8  3.78
           weight
Mix6        0.412
Mix1        0.257
Mix2        0.167
Mix.global  0.102
```

```
MixNull     0.062
Models ranked by AICc(x)
Random terms (all models):
'1 | Group/ID.Ind'


> ### effect size on the basis of full model (Mix.global)
> summary(Mix.global)
Generalized linear mixed model fit by maximum likelihood
  (Laplace Approximation) [glmerMod]
 Family: binomial  ( logit )
Formula:
MultipleInfec ~ Provisioning + Antiparasitic + FecalSampleW + (1
| Group/ID.Ind)
   Data: MultINF

     AIC      BIC   logLik deviance df.resid
   192.7    219.9    -90.4    180.7      681

Scaled residuals:
    Min      1Q  Median      3Q     Max
-0.2898 -0.2360 -0.1390 -0.1317 10.0038

Random effects:
 Groups       Name        Variance Std.Dev.
 ID.Ind:Group (Intercept) 0        0
 Group        (Intercept) 0        0
Number of obs: 687, groups:  ID.Ind:Group, 31; Group, 2

Fixed effects:
                 Estimate Std. Error z value Pr(>|z|)
(Intercept)       -4.0699     1.2922  -3.150  0.00163 **
ProvisioningLOW    1.1663     0.4911   2.375  0.01756 *
AntiparasiticNT    0.5515     0.5662   0.974  0.33002
FecalSampleW      -0.1787     0.4076  -0.438  0.66114
---
Signif. codes:  0 '***' 0.001 '**' 0.01 '*' 0.05 '.' 0.1 ' ' 1

Correlation of Fixed Effects:
            (Intr) Provis Antipar
ProvisionLOW -0.260
AntiparNT    -0.407  0.067
FecalSampleW -0.872 -0.037  0.041


 > confint(Mix.global)
 Computing profile confidence intervals ...
                      2.5 %     97.5 %
 .sig01            0.0000000  1.0946245
 .sig02            0.0000000  0.7931448
 (Intercept)      -7.0479044 -1.8402247
 ProvisioningLOW   0.2491723  2.2106328
 AntiparasiticNT  -0.4662928  1.8128465
 FecalSampleW     -0.8876707  0.7615993
```

**GLMM full outputs for Tables 3 and 4. Model selection and model averaging for individual body weight's variation.**

Response variable: weights (individual body weights). Fixed effects= Antiparasitic [levels = AntiparasiticNT (Not Treated), AntiparasiticT (Treated)], Provisioning [levels = ProvisioningLOW (low regime), ProvisioningHIGH (high regime)], Sex [levels = SexM (males), SexF (females)]. Random effects = Group, ID. Ind (individuals' identities).

```
> library(MuMIn)
>
> options(na.action = "na.fail")
> mod.global<-lme(weights~ Antiparasitic + Provisioning + Sex, data=subtable,
random=~1|Group/Individual,method="ML")
> summary(mod.global)
Linear mixed-effects model fit by maximum likelihood
 Data: subtable
       AIC      BIC    logLik
  17.01386 31.06519 -1.50693

Random effects:
 Formula: ~1 | Group
        (Intercept)
StdDev:    0.165035

 Formula: ~1 | Individual %in% Group
        (Intercept)  Residual
StdDev:   0.1265117 0.2209758

Fixed effects: weights ~ Antiparasitc + Provisioning + Sex
                 Value  Std.Error DF   t-value p-value
(Intercept)   2.5850626 0.15064553 44 17.159902  0.0000
AntiparNT    -0.0401147 0.06815837 44 -0.588551  0.5592
ProvisionLOW -0.2086297 0.06774519 44 -3.079624  0.0036
SexM          1.1105414 0.13279197  6  8.363016  0.0002
 Correlation:
            (Intr)   Antipar Provis
AntiparNT     -0.288
ProvisionLOW -0.308  0.289
SexM         -0.252 -0.043   -0.029

Standardized Within-Group Residuals:
       Min         Q1        Med         Q3        Max
-2.0202258 -0.6544378 -0.1087115  0.6359339  1.9616382

Number of Observations: 55
Number of Groups:
                Group Individual %in% Group
                    2                     9
>
> MMI3.1<-dredge(mod.global,rank="AICc")
> MMI3.1
Global model call: lme.formula(fixed = weights ~ Antiparasitic + Provisioning
+ Sex, data = subtable,
    random = ~1 | Group/Individual, method = "ML")
---
Model selection table
  (Intrc) Antip  Provi Sex df  logLik AICc delta weight
7   2.560            +   +  6  -1.689 17.1  0.00  0.728
8   2.585     +      +   +  7  -1.507 19.4  2.27  0.234
5   2.453                +  5  -6.194 23.6  6.48  0.028
6   2.442     +          +  6  -6.137 26.0  8.90  0.009
3   2.879            +      5 -10.621 32.5 15.34  0.000
4   2.912     +      +      6 -10.313 34.4 17.25  0.000
1   2.772                   4 -14.778 38.4 21.23  0.000
2   2.769     +             5 -14.773 40.8 23.64  0.000
Models ranked by AICc(x)
Random terms (all models):
'1 | Group', '1 | Individual %in% Group'

>
> # select models 95% cumulative weight criteria
> output <- subset(MMI3.1, cumsum(MMI3.1$weight) <= .95)
> output
Global model call: lme.formula(fixed = weights ~ Antiparasitic + Provisioning
+ Sex, data = subtable,
    random = ~1 | Group/Individual, method = "ML")
---
Model selection table
```

```
  (Intrc)   Provi Sex df logLik AICc delta weight
7    2.56     +    +  6 -1.689 17.1     0      1
Models ranked by AICc(x)
Random terms (all models):
'1 | Group', '1 | Individual %in% Group'


> ### effect size on the basis of full model

> mod.global <-lme(weights~Antiparasitic + Provisioning + Sex, da
ta=subtable,random=~1|Group/Individual,method="ML")
> summary(mod global)
Linear mixed-effects model fit by maximum likelihood
 Data: subtable
       AIC      BIC   logLik
  17.01386 31.06519 -1.50693

Random effects:
 Formula: ~1 | Group
        (Intercept)
StdDev:    0.165035

 Formula: ~1 | Individual %in% Group
        (Intercept)  Residual
StdDev:   0.1265117 0.2209758

Fixed effects: weights ~ Antiparasitic + Provisioning + Sex
                 Value  Std.Error DF   t-value p-value
(Intercept)       2.5850626 0.15064553 44 17.159902  0.0000
AntiparasiticNT  -0.0401147 0.06815837 44 -0.588551  0.5592
ProvisioningLOW  -0.2086297 0.06774519 44 -3.079624  0.0036
SexM              1.1105414 0.13279197  6  8.363016  0.0002
 Correlation:
                 (Intr) Antip Provis
AntiparasiticNT  -0.288
ProvisioningLOW  -0.308  0.289
SexM             -0.252 -0.043 -0.029

Standardized Within-Group Residuals:
       Min         Q1        Med         Q3        Max
-2.0202258 -0.6544378 -0.1087115  0.6359339  1.9616382

Number of Observations: 55
Number of Groups:
                Group Individual %in% Group
                    2                     9


>
```

**Table S6. Identity, age-sex classes and date of treatment for the individuals that were treated with antiparasitic drugs during the two winters of the experiment.**

| Year | Group | Treated individual | Age-sex class | Date of antiparasitic treatment |
|---|---|---|---|---|
| Winter 2013 | MACUCO | OFE | ADF | June 17th, 2013 |
| | | EST | ADF | June 28th, 2013 |
| | | ING | ADF | July 2nd, 2013 |
| | | THE | ADF | June 28th, 2013 |
| | | EDU | ADM | June 18th, 2013 |
| | | SRG | ADM | June 18th, 2013 |
| | | TIT | ADM | June 18th, 2013 |
| | SPOT | JOS | ADF | June 27th, 2013 |
| | | EVA | ADF | June 27th, 2013 |
| | | TET | ADM | June 27th, 2013 |
| Winter 2014 | MACUCO | CRD | ADF | July 16th, 2014 |
| | | MAW | ADF | July 16th, 2014 |
| | | SOL | ADF | July 16th, 2014 |
| | | ERN | ADM | July 16th, 2014 |
| | | RIC | SUBM | July 16th, 2014 |
| | SPOT | BIA | ADF | July 16th, 2014 |
| | | DAN | ADF | July 16th, 2014 |
| | | RIN | ADM | July 18th, 2014 |
| | | TRU | ADM | July 18th, 2014 |

ADF = adult female; ADM = adult male; SUBM = subadult male.

Figure S1. Methods used for recording body weight and provisioning capuchin groups. Panel (a) shows the researcher IA recording body weight of a female capuchin monkey. The scale was mounted on a tree trunk and baited with smashed bananas. When a capuchin was fully supported on the balance, an observer looked at the remote digital display and recorded the individual's weight. Panel (b) shows the platforms used for supplying capuchin monkeys with bananas. These wooden platforms were raised up on the tree branches by researchers as soon as the group arrived at a provisioning site.

(a) (b)

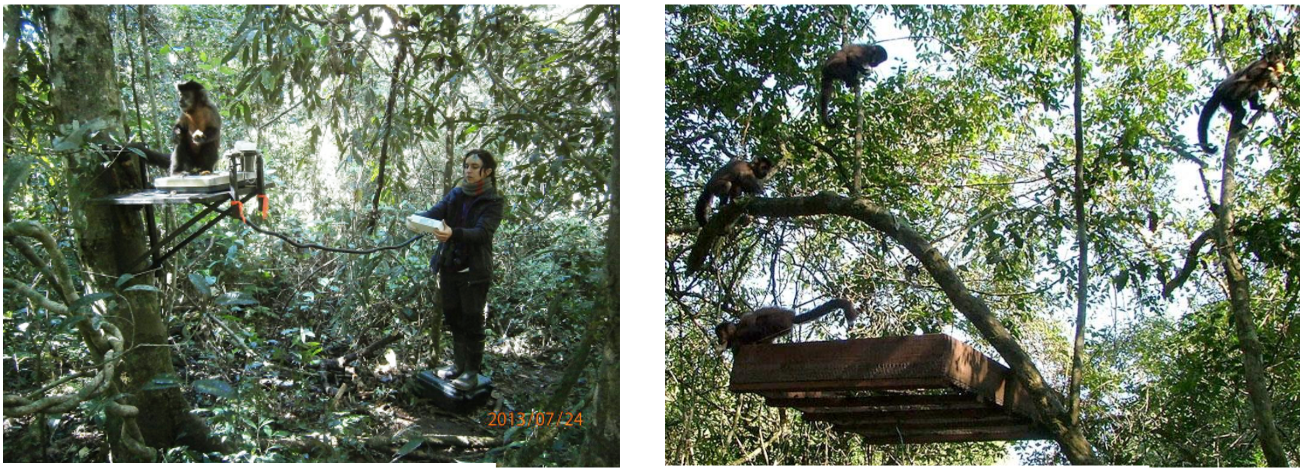


Photo: Melisa Unger

Photo: Brandon Wheeler

<u>Details of the modified cage used to temporarily trap selected individuals and supply the antiparasitic treatment.</u>

We controlled individual access to the cage remotely with a system of ropes: pulling rope #1 enabled us to close the main entrance door when the selected individual was already inside the outer cage's compartment, pulling rope #2 allowed the opening of the additional inner door that let the monkey reach the inner compartment with the banana piece, and pulling rope #3 permitted the opening of the main cage's entrance to ensure the monkey the way out after the consumption of the whole banana piece. This procedure ensured that only one individual at a time could enter the cage and allowed us to select the

individuals that should access the anti-parasitic drugs. In order to keep a distance from the selected subject, we positioned the cage on a platform that we raised upon a branch tree (see the description of the provisioning platform in Methods section). Then, we were able to handle the cage ropes from the ground while the individual approached the cage.

Fig. S2. (a) The modified Tomahawk cage we used to trap some individuals to administer the antiparasitic drugs. (b) The platform upon which we set the cage and one capuchin individual approaching it.

(a) (b)

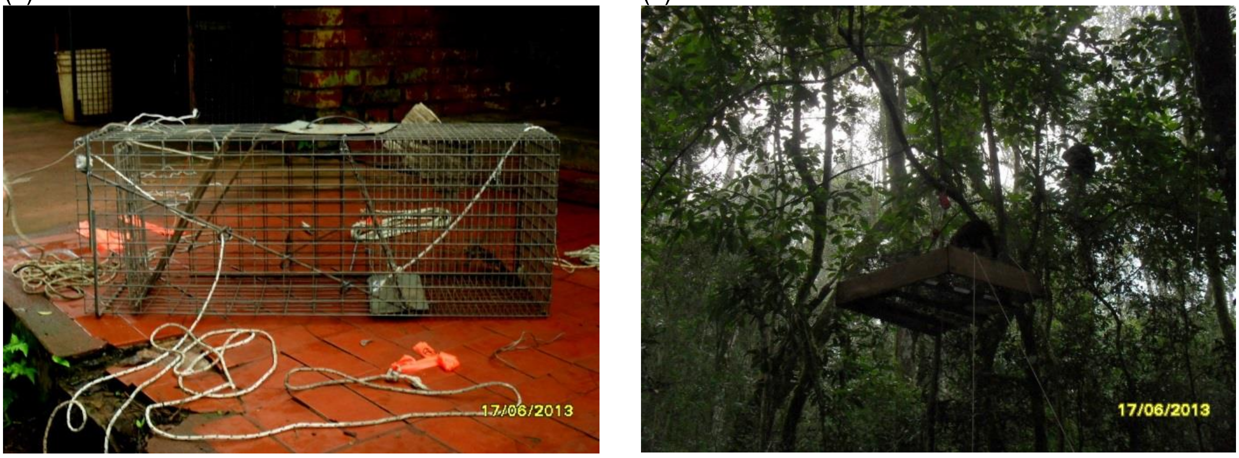

Fig. S3. A diagram of the experimental design used in this study. Different combinations of treatments of provisioning regimes and anti-parasitic drugs supply for the Macuco group (left) and the Spot group (right), during winter 2013 (top) and winter 2014 (down). A- F- = Anti-parasitic-Not Treated and Low food Provisioning (= Control); A+ F- = Anti-parasitic-Treated and Low food Provisioning; A- F+ = Anti-parasitic - Not Treated and High food Provisioning; A+ F+ = Anti-parasitic -Treated and High food Provisioning.

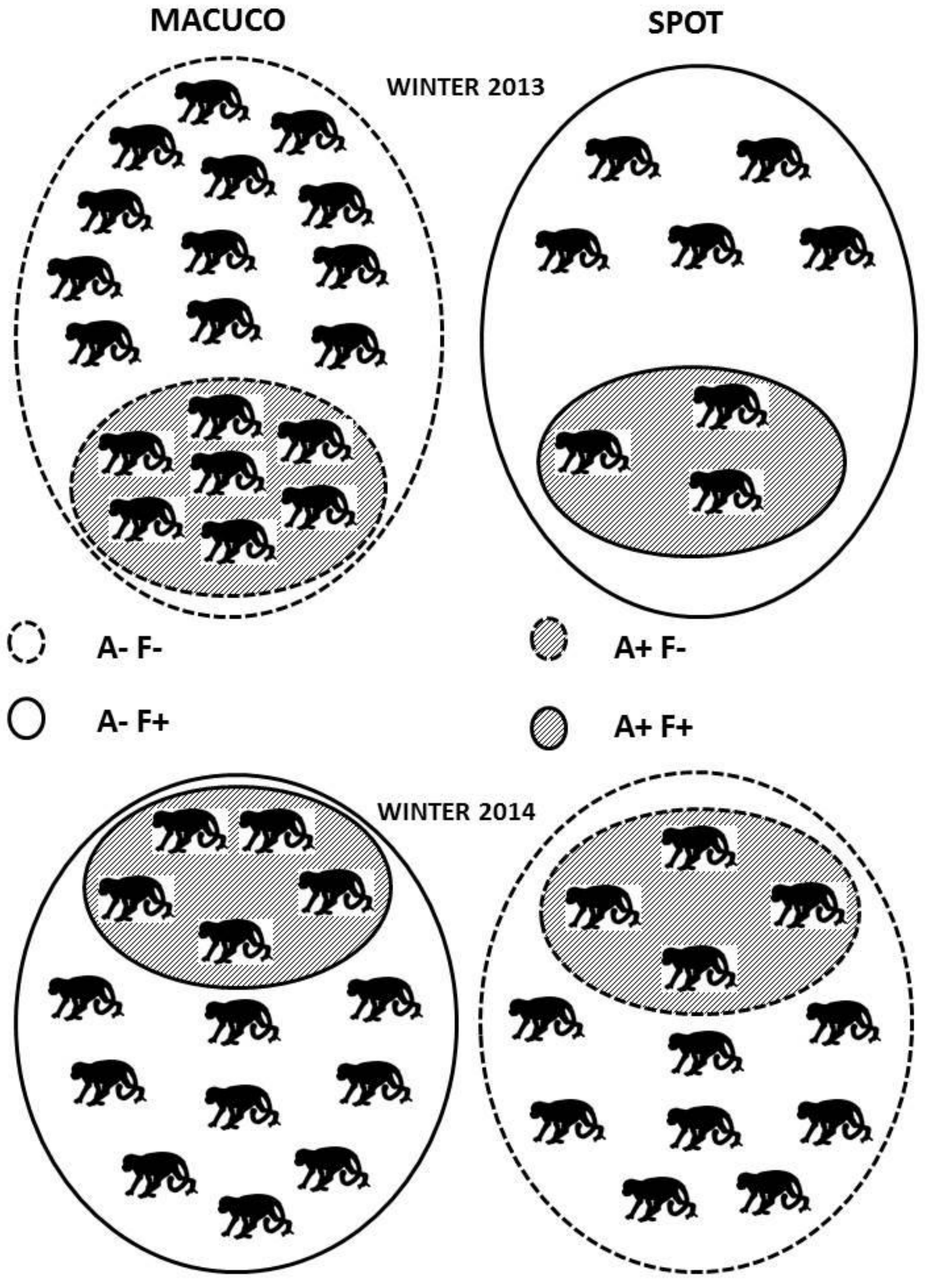

Supplement: Supplementary file 1 — Supplementary Material [file 41598_2017_12803_MOESM1_ESM.pdf]
